# Melatonin Augments the Expression of Core Transcription Factors in Aged and Alzheimer’s Patient Skin Fibroblasts

**DOI:** 10.3390/biology13090698

**Published:** 2024-09-05

**Authors:** Mayuri Shukla, Raphiporn Duangrat, Chutikorn Nopparat, Areechun Sotthibundhu, Piyarat Govitrapong

**Affiliations:** 1 Chulabhorn Graduate Institute, Chulabhorn Royal Academy, Kamphaeng Phet 6, Bangkok 10210, Thailand; 2 Innovative Learning Center, Srinakharinwirot University, Sukhumvit 23, Bangkok 10110, Thailand; 3 Chulabhorn International College of Medicine, Thammasat University, Pathumthani 12120, Thailand

**Keywords:** Alzheimer’s disease, fibroblasts, melatonin, core transcription factors, Sox2, Oct4, Nanog

## Abstract

**Simple Summary:**

Alzheimer’s Disease is the most common form of dementia. The disease is progressive and irreversible in nature, leading to reduced neurogenesis, neuronal loss, and cognitive decline. Mounting evidence suggests the crucial role of transcription factors in the regulation of the initiation and progression of Alzheimer’s disease. Sox2, Oct4, and Nanog are such pivotal transcription factors that are critically involved in neurogenesis, AD pathology, and aging. It is critical to detect early pathological alterations; therefore, the use of peripheral tissues like skin fibroblasts are recommended for investigative purposes. Our study demonstrated that fibroblasts from Alzheimer’s patients and aged candidates present alterations in the expression of these transcription factors. Strategies to enhance Sox2, Oct4, and Nanog expression levels may be used as a novel intervention for reducing amyloid beta neurotoxicity in the AD brain, preventing senescence and delaying aging. Henceforth, we explored the effect of melatonin administration on these factors. Melatonin enhanced the expression of these transcription factors in the Alzheimer’s and aged group, suggesting a prompt therapeutic strategy against neurodegenerative processes and aging along with boosting the regenerative capacity of cells.

**Abstract:**

Alzheimer’s disease (AD) is a progressive neurodegenerative disorder. Altered neurogenesis and the appearance of AD pathological hallmarks are fundamental to this disease. SRY-Box transcription factor 2 (Sox2), octamer-binding transcription factor 4 (Oct4), and Nanog are a set of core transcription factors that play a very decisive role in the preservation of pluripotency and the self-renewal capacity of embryonic and adult stem cells. These factors are critically involved in AD pathogenesis, senescence, and aging. Skin fibroblasts are emblematic of cellular damage in patients. We, therefore, in the present study, analyzed the basal expression of these factors in young, aged, and AD fibroblasts. AD fibroblasts displayed an altered expression of these factors, differing from aged and young fibroblasts. Since melatonin is well acknowledged for its anti-aging, anti-senescence and anti-AD therapeutic benefits, we further investigated the effects of melatonin treatment on the expression of these factors in fibroblasts, along with precise validation of the observed data in human neuroblastoma SH-SY5Y cells. Our findings reveal that melatonin administration augmented the expression levels of Sox2, Oct4, and Nanog significantly in both cells. Altogether, our study presents the neuroprotective potential and efficacy of melatonin, which might have significant therapeutic benefits for aging and AD patients.

## 1. Introduction

Alzheimer’s disease (AD) is a chronic neurodegenerative brain disorder affecting people in the millions worldwide, and this number is anticipated to double in a few decades, with prevalence found largely among the elderly population of 60 years of age and above. The extracellular deposition of amyloid beta (Aβ) and intracellular accumulation of hyperphosphorylated tau protein mark the characteristic traits of this debilitating disorder that primarily impacts the prefrontal cortex and hippocampus in the brain, eventually causing a progressive decline in learning and memory with substantial neuronal loss [1]. The pathophysiological processes in AD develop decades before the symptoms are evinced. Therefore, early clinical biomarkers with reliability and accuracy are needed to diagnose this disease, which requires a multidisciplinary therapeutic regime for prompt prognosis.

Investigation using peripheral cells aids in understanding the primary pathophysiological mechanisms leading to neurodegenerative diseases. In this frame of reference, several studies have emphasized the use of peripheral tissues as a potential diagnostic approach, where human skin fibroblasts have been extensively investigated to study pathophysiological abnormalities related to psychiatric [2], neurodegenerative diseases, like Parkinson’s disease [3,4], Huntington’s disease [5], amyotrophic lateral sclerosis [6], and AD [7]. This model could prove to be a potential tool to study and investigate the pathological confrontations in diseases like AD [8,9].

Importantly, adult neurogenesis tends to decline age-dependently, and aging is the major risk factor for AD, where decreased neurogenesis tremendously affects the cognitive health span [10], accounting for the memory deficits featured in AD patients [11]. The pluripotency and differentiation states of embryonic stem cells are regulated by a set of core transcription factors, primarily SRY-Box transcription factor 2 (Sox2), octamer-binding transcription factor 4 (Oct4), and Nanog, which have been acknowledged as the core pluripotency network acting jointly to activate genes that sustain a stem-like state in embryonic stem cells, maintaining self-renewal, directing potentiation of the cells into neural precursor cells, and retaining the capacity to proliferate in those cells [12]. Proliferation of neural precursor cells was considered important for the preservation of neuronal populations and the compensation of the abolished cells in AD, as Aβ oligomers exhibit suppressive activity against cell proliferation and inhibit the generation of neural precursor cells [13]. Not only could they retain cell stemness, but their expressions were also largely implicated in the attempt to instigate fibroblast-derived cell reprogramming in the treatment of AD patients [14], which is an ongoing area of research. Imperatively, studies have found that each of the factors are individually involved in AD pathogenesis and in the prevention of the disease’s progression. For instance, the downregulation of Sox2 expression is known to trigger neurodegeneration, as it is crucially involved in neurogenesis during normal aging and in AD [15,16]. Moreover, the deletion of its expression also leads to the instigation of cellular senescence and premature aging [17]. Concerning Oct4, it has been shown that its expression in endothelial cells protects cells from DNA damage and replicative senescence [18], and the embryonic stem cell gene Nanog has been found to protect against neuronal Aβ toxicity and oxidative stress [19]. Moreover, it has the capacity to delay aging with the potential of reversing it, which is the reason research is being emphasized on identifying agents that can enhance its expression.

Discoveries regarding the roles of melatonin, a pineal gland neuro-hormone, as a regulator of circadian rhythm, aging, Aβ plaque and neurofibrillary formation, etc., has emphasized the importance of melatonin as an alternative prevention of AD incidence [20]. More recent evidence has established a convergence between melatonin and stem cell therapy. Apart from the functions mentioned above, melatonin was observed to be a critical participant in the process of neurogenesis of the neural precursor cells that possess multipotency as well as promoting neural proliferation [21,22]. Concomitantly, melatonin was reported to improve the efficiency of somatic cell reprogramming into neurons [23]. Fibroblasts provide an exclusive rationale and opportunity where findings from cells can be translated to human subjects; therefore, in the present study, we attempt to investigate the effect of melatonin administration on the expression of the core transcription factors Sox2, Oct4, and Nanog in human skin fibroblasts derived from young, aged, and AD human subjects and affirmed the findings in neuronal cells with significant correlation.

## 2. Material and Methods

### 2.1. Materials

Minimum essential medium (MEM), Ham’s F-12 medium, fetal bovine serum (FBS), penicillin, and streptomycin were purchased from Gibco BRL (Gaithersburg, MD, USA). Melatonin and luzindole were obtained from Sigma-Aldrich (St Louis, MO, USA). Rabbit polyclonal anti-Sox2 antibody and mouse anti-Nanog were purchased from EMD Millipore (Temecula, CA, USA). Mouse anti-Actin was purchased from Millipore (Billerica, MA, USA). Mouse anti-Oct3/4 (C-10) was supplied by Santa Cruz Biotechnology (Dallas, TX, USA). Luminata™ classico and crescendo (HRP substrate) were purchased from Millipore (Billerica, MA, USA). Aβ42 was purchased from Anaspec (Fremont, CA, USA).

### 2.2. Fibroblast Cell Culture and Treatment

Young and aged human skin fibroblasts were acquired from the NIGMS Human Genetic Cell Repository, and Alzheimer’s patient skin fibroblasts were obtained from the NIA Aging Cell Culture Repository (Camden, NJ, USA). Cells were grown in 75 cm^2^ (T-75) and 25 cm^2^ (T-25) flasks in a complete media (a mixture of DMEM and F-12 in a 1:1 proportion with an addition of 15% non-inactivated FBS, 10 units/mL of penicillin, and 10 μg/mL of streptomycin) and maintained in a 95% humidified air incubator at a temperature of 37 °C and 5% CO_2_. Cells were seeded in 60 mm Petri dishes according to the required number of experimental groups. Cells were pre-treated with 1% FBS media containing various concentrations of freshly prepared melatonin (0.01, 0.1, 1, and 10 μM) for 24 h.

### 2.3. SH-SY5Y Cell Culture

Human dopaminergic neuroblastoma SH-SY5Y cells were obtained from the American Type Culture Collection (Manassas, VA, USA). Cells were grown in a 75 cm^2^ tissue culture flask (T-75) containing complete media (mixing of MEM and F-12 in a 1:1 proportion with addition of 10% inactivated FBS and 100 units/mL of penicillin, and 10 μg/mL of streptomycin) and maintained in a 95% humidified air incubator at a temperature of 37 °C and 5% CO_2_. SH-SY5Y cells were seeded in 60 mm Petri dishes and allowed to grow until they reached 80% confluence. To examine the effect of different concentrations of melatonin or Aβ, cells were then treated with and without various concentrations of melatonin (1 and 10 µM) or Aβ (0.1, 1, and 2 µM) in media containing 1% FBS for 24 h. After the optimal concentrations of melatonin and Aβ were determined, the cells were treated with 1 µM Aβ with and without 10 µM melatonin, or were pretreated with 10 µM luzindole, a melatonin receptor antagonist, prior to 1 h of 10 µM melatonin treatment. Next, cells were incubated in the absence or presence of Aβ42 (1 µM), dissolved in 1% FBS media for 24 h before cell collection.

### 2.4. Assessment of the Effects of Melatonin on Cell Viability of Aβ-Treated SHSY-5Y Cells and on AD Fibroblast

Muse^®^ Count & Viability Kit (MCH100102, MerckMillipore, Guyancourt, France) was used to assess the toxicity of Aβ and the protective effects of melatonin on cell viability. After treatment of cells described prior, cells were collected by trypsinization and pelleted by centrifugation at 1000 rpm for 5 min at 4 °C, then diluted in the Muse^®^ Count & Viability Kit reagent according to the manufacturer’s instructions. After a 5 min incubation at room temperature, samples were analyzed in a Guava^®^ Muse^®^ Cell Analyzer (Luminex Corporation, Austin, TX, USA), a flow cytometry-based technology, at 5000 events per sample to calculate percent viability. The results were collected and analyzed by Muse 1.9 analysis software.

### 2.5. Western Blot Analysis

Cells were collected and lysed with RIPA buffer (150 mM NaCl, 50 mM Tris-base, 1 mM Phenyl methanesulfonyl fluoride (PMSF), 1 mM EDTA, 1% Triton X-100, 0.5% sodium deoxycholate, 0.1% SDS, 1% protease inhibitor, and 1% phosphatase inhibitor) and sonicated for 30 s twice. Cell lysates were centrifuged at 12,000× *g* for 15 min at 4 °C and the supernatant collected for western blot analysis. Protein concentrations were determined by Bradford protein assay. Samples were electrophoresed on 12–17.5% SDS–PAGE gels and transferred onto PVDF membranes (Amersham Biosciences, Piscataway, NJ, USA). The transfer efficacies were evaluated by staining the membrane with Ponceau Red solution (Bio-Rad Laboratories, Hercules, CA, USA). The PVDF membranes were then blocked with blocking solution (5% non-fat milk or 3% BSA in TBS containing 0.1% Tween-20, TBS-T) for 1 h at room temperature and then incubated overnight at 4 °C with the primary antibody. After washing three times with TBS-T, the membranes were incubated with a horseradish-conjugated anti-mouse or anti-rabbit IgG for 90 min. The signals on the membranes were detected using Luminata™ classico and crescendo (HRP substrate, Millipore Corporation, Billerica, MA, USA) reagents. The immunoblots were quantified by measuring the density of each band using densitometry analysis with the Image J program version ImageJ 1.53e (National Institutes of Health, Bethesda, MD, USA)

### 2.6. Immunocytochemistry-Staining

SH-SY5Y cells were incubated for 24 h in a 24-well plate containing complete media and a coverslip precoated with poly-L-ornithine, allowing cells to grow and attach onto the coverslip. The cells were then either incubated with (1) 1 µM melatonin serum-free media alone, (2) 1 µM Aβ42 in serum-free media alone, or (3) 1 µM melatonin in serum-free media for 2 h before 1 µM Aβ42 treatment for another 24 h in a 95% humidified air incubator at a temperature of 37 °C and 5% CO_2_. After that, coverslips with attached cells were immersed in 4% paraformaldehyde in 0.1 M phosphate-buffered saline (PBS) for 20 min at room temperature followed by washing with PBS 3 times before gently shaking with blocking buffer (0.1 M PBS, 0.1% Triton X-100, and 5% normal goat serum (NGS)) for 1 h at room temperature. Then, the coverslips were incubated with the primary antibody rabbit anti-Sox2 (1:200) diluted in 0.1 M PBS +5% NGS at 4 °C overnight followed by incubation with Alexa 488-conjugated anti-rabbit (1:400) secondary antibodies at room temperature for 1 h then washed with PBS and mounted with antifade reagent (Vectashield, Vector Laboratories, Burlingame, CA, USA) before being visualized under the microscope. The staining of Sox2 was visualized by a confocal laser scanning microscope (FV 3000, Olympus, Singapore).

### 2.7. Statistical Analysis

One-way analysis of variance (one-way ANOVA) and Tukey’s post-hoc test were used to analyze the statistical differences among groups, in which a *p*-value < 0.05 was considered significant. Statistics calculations were performed using GraphPad Prism7 (Boston, MA, USA). The data were expressed as mean ± SEM.

## 3. Results

### 3.1. Cell Viability in AD Fibroblast or in Aβ-Treated SH-SY5Y Cells Rescued by Melatonin Treatment

**Figure 1:**
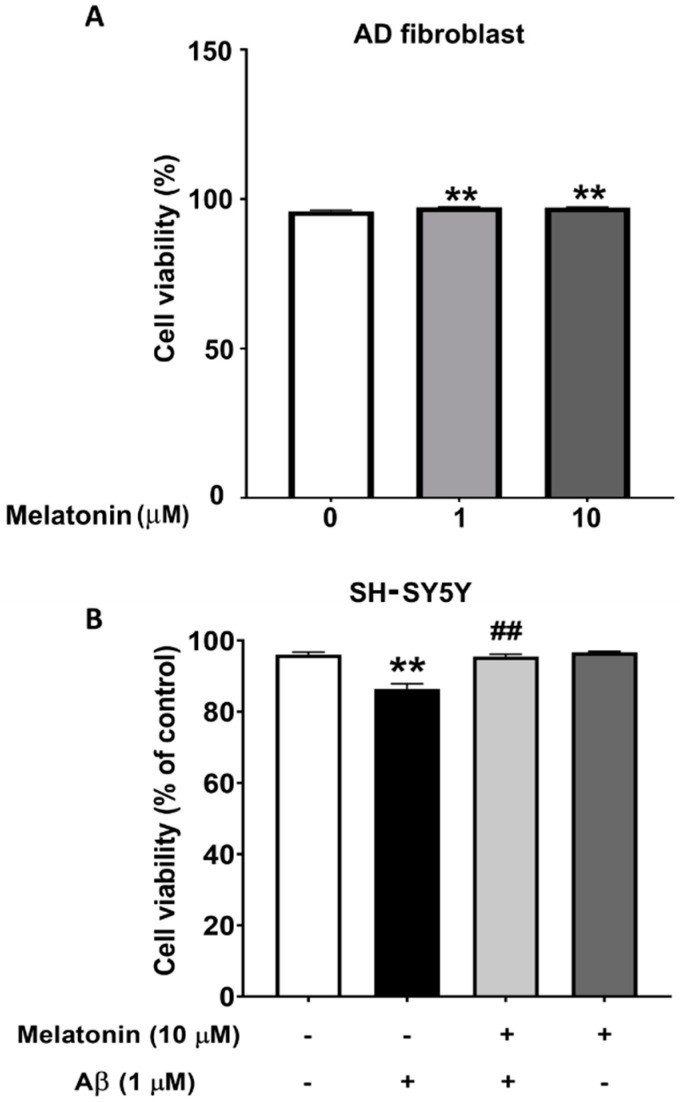
The effect of melatonin on the cell viability of AD fibroblasts after addition of 1 µM and 10 µM melatonin for 24 h (**A**). The effect of melatonin on the cell viability of Aβ-treated SH-SY5Y cells after addition of 1 µM melatonin for 24 h (**B**). The results were collected and analyzed by Muse 1.9 analysis software. Data are expressed as mean ± SEM calculated using one-way ANOVA and Tukey’s post hoc test with significant difference at ** = *p* < 0.01 when compared to untreated controls, and ## = *p* < 0.01 when compared to Aβ-treated SH-SY5Y cells *(n* = 4).

To demonstrate that Aβ is toxic and that melatonin has a protective effect on cells, the viability of SH-SY5Y cells were assessed. The results from the MUSE count and viability assay show that cells treated with Aβ for 24 h have a statistically significant decrease to 93% (*p* < 0.01) in cell viability compared to controls, indicating a toxic effect. Cells pretreated with melatonin prior to Aβ addition displayed a statistically significant increase to 98% (*p* < 0.01) in cell viability compared to the Aβ-treated group. The results demonstrate that Aβ reduces cell viability, and pretreatment with melatonin can attenuate this decrease (Figure 1B). We also determined the effect of melatonin on the cell viability of AD fibroblasts. After adding melatonin at 1 µM and 10 µM for 24 h, cell viability significantly increased to 97% (*p* < 0.01) compared to the untreated control group (Figure 1A).

### 3.2. Basal Levels of Core Transcription Factors in Young, Aged, and Alzheimer’s Skin Fibroblasts

**Figure 2:**
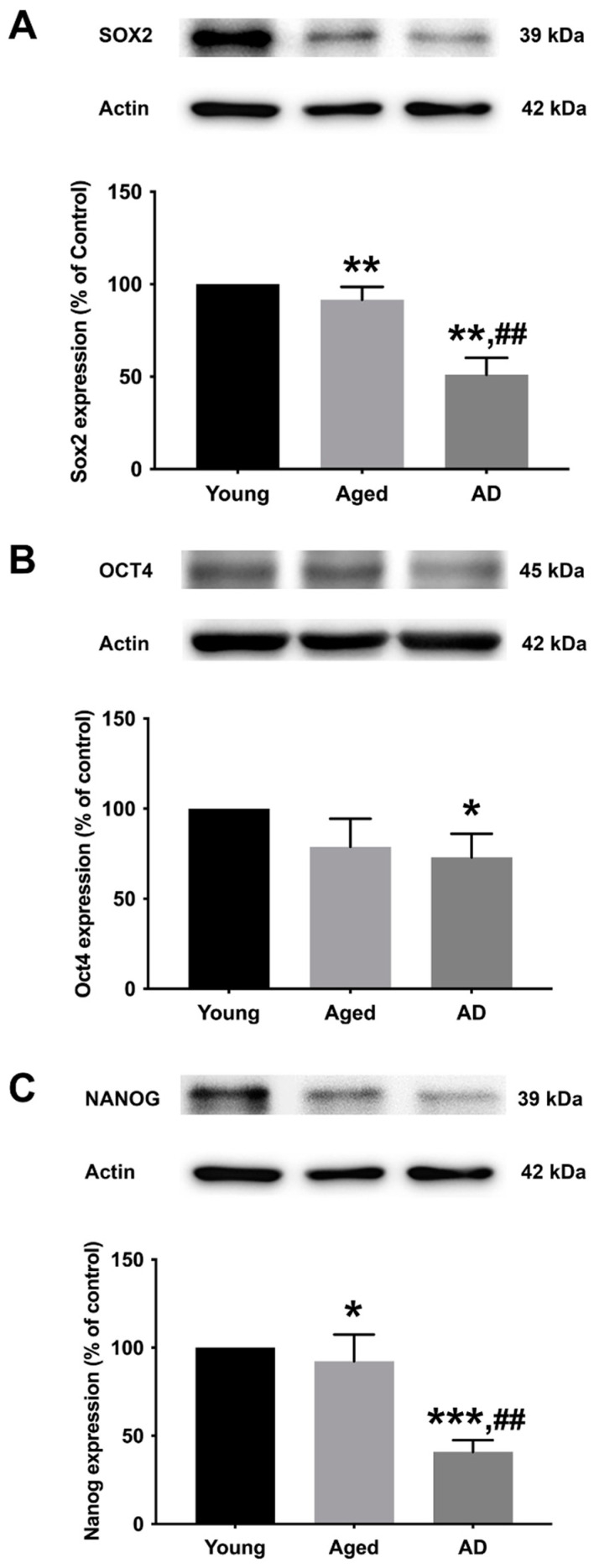
The expression levels of (**A**) Sox2, (**B**) Oct4, and (**C**) Nanog in young, aged, and Alzheimer’s skin fibroblasts. Young, aged, and Alzheimer’s skin fibroblasts were cultured in serum-free media for 24 h before being analyzed for Sox2, Oct4, and Nanog expression levels via Western blotting with actin as a loading control. Results are reported as percentage of the young fibroblast. Data are expressed as mean ± SEM with significant difference at * = *p* < 0.05, ** = *p* < 0.01, and *** = *p* < 0.001 of aged or Alzheimer’s groups compared to young control groups and at ## = *p* < 0.01 of Alzheimer’s compared with aged groups. Data were calculated using one-way ANOVA and Tukey’s post hoc test (*n* = 3).

To determine the preexisting levels of Sox2, Oct4, and Nanog in young, aged, and Alzheimer’s human skin fibroblasts, the three types of fibroblasts were cultured in serum-free media for 24 h before expression analysis with Western blotting (Figure 2A–C, Supplementary Figure S1). The results illustrated a significant decrease in all marker levels (Sox2, Oct4, and Nanog) in Alzheimer’s fibroblasts compared to the young control group. The observed expression levels of Sox2 and Nanog in the aged group were significantly lower compared to the young group. Moreover, the levels of Sox2 and Nanog in AD fibroblasts were found to be significantly lower compared to the aged group.

### 3.3. Concentration-Dependent Effect of Melatonin on the Core Transcription Factors in Young Skin Fibroblasts

**Figure 3:**
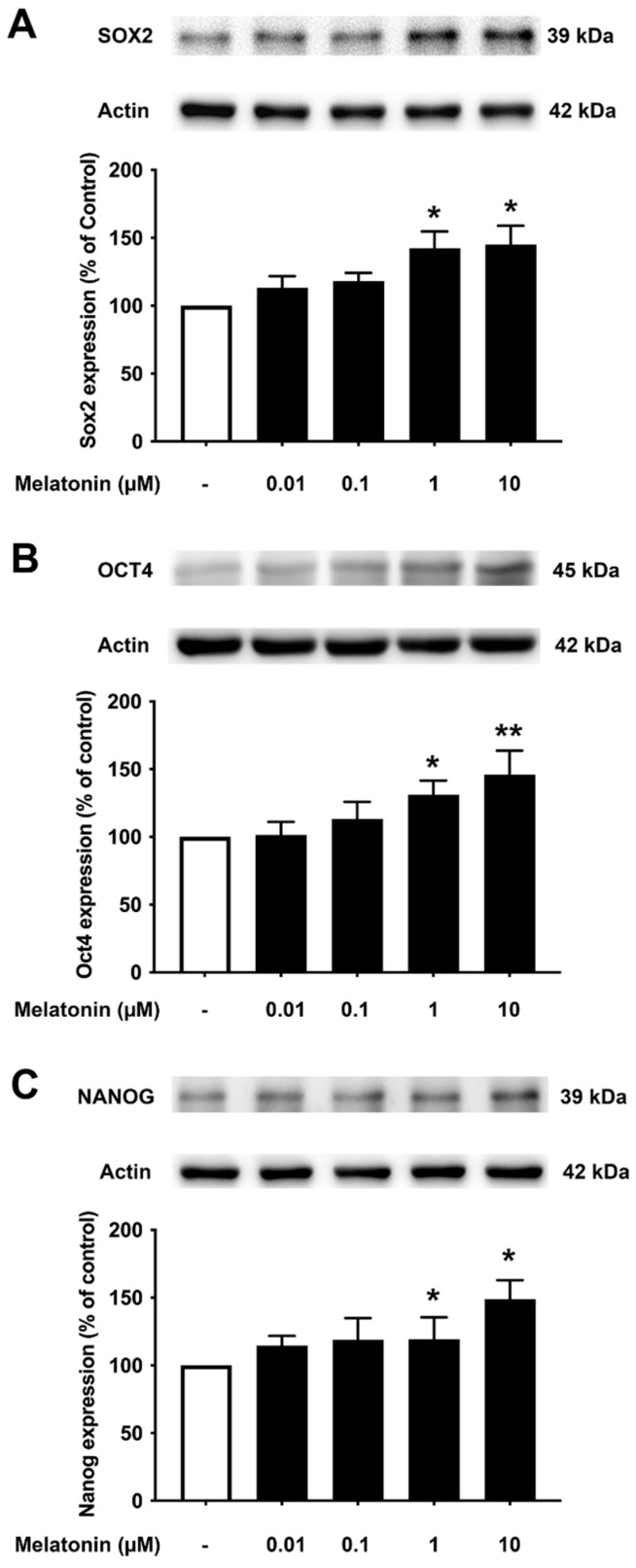
The effect of various concentrations of melatonin on (**A**) Sox2, (**B**) Oct4, and (**C**) Nanog expression levels in young skin fibroblasts. Young skin fibroblasts were incubated in various (0.01, 0.1, 1, 10 µM) concentrations of melatonin in serum-free media for 24 h. Control cells were cultured in serum-free media for 24 h. Western blotting was used for the analysis of protein expression levels with actin as a loading control. Results are reported as a percentage of the control. Data are expressed as mean ± SEM calculated using one-way ANOVA and Tukey’s post hoc test with significant difference at * = *p* < 0.05 and ** = *p* < 0.01 when compared to untreated controls (*n* = 4).

To determine the effect of different concentrations of melatonin on Sox2, Oct4, and Nanog in young fibroblasts, cells were treated with varying concentrations of melatonin (0.01, 0.1, 1, 10 μM, and 0 μM for controls) for 24 h, and protein expression levels were detected using Western blotting. The results demonstrated that melatonin increased the expression levels of Sox2, Oct4, and Nanog in a concentration-dependent manner with significant inductions at 1 and 10 μM compared to untreated controls (Figure 3A–C, Supplementary Figure S2).

### 3.4. Concentration-Dependent Effect of Melatonin on the Core Transcription Factors in Aged Fibroblasts

**Figure 4:**
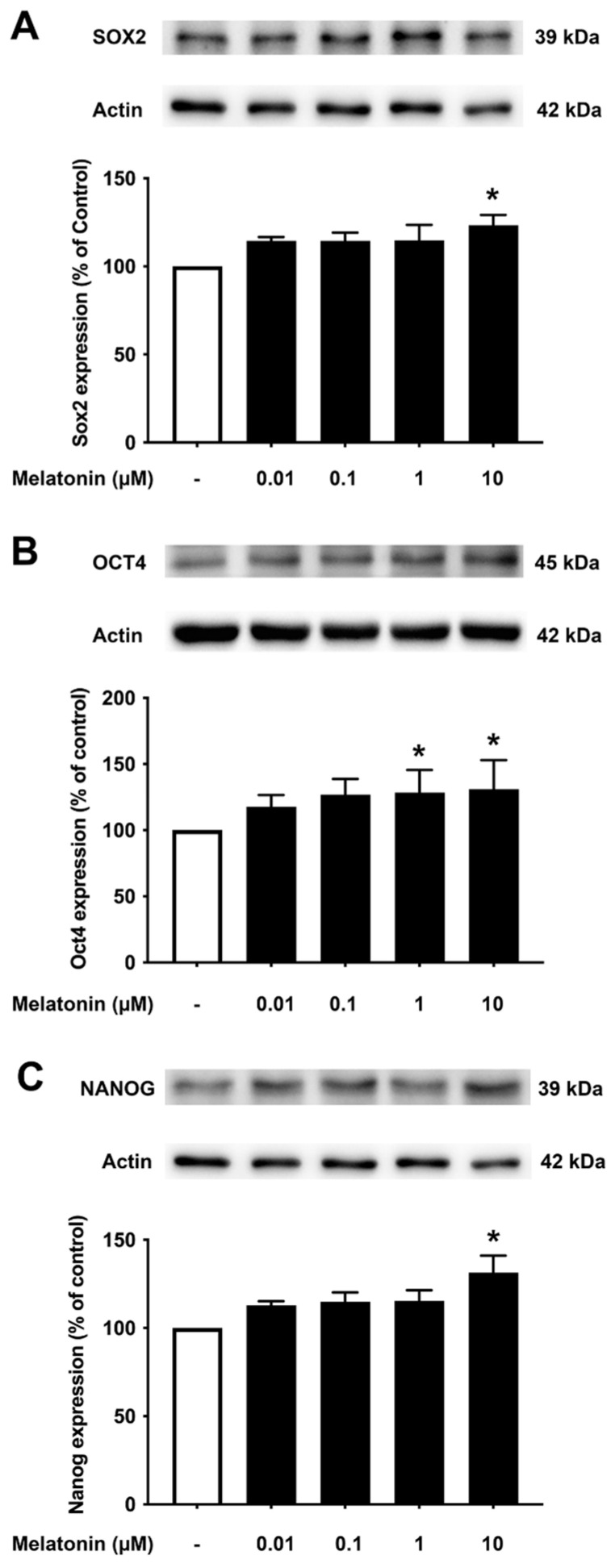
The effect of various concentrations of melatonin on (**A**) Sox2, (**B**) Oct4, and (**C**) Nanog expression levels in aged skin fibroblasts. Aged skin fibroblasts were incubated in various (0.01, 0.1, 1, 10 µM) concentrations of melatonin in serum-free media for 24 h. Control cells were cultured in serum-free media for 24 h. Western blotting was used for the analysis of protein expression levels with actin as a loading control. Results are reported as a percentage of the control. Data are expressed as mean ± SEM calculated using one-way ANOVA and Tukey’s post hoc test with significant difference at * = *p* < 0.05 when compared to untreated controls (*n* = 4).

To investigate the effect of different concentrations of melatonin on Sox2, Oct4, and Nanog in aged skin fibroblasts, cells were treated with various concentrations of melatonin (0.01, 0.1, 1, 10 μM, and 0 μM for controls) for 24 h, and protein expression levels were determined using Western blotting. The results demonstrated that 10 μM melatonin significantly increased the expression of Sox2, Oct4, and Nanog compared to untreated controls (Figure 4A–C, Supplementary Figure S3).

### 3.5. Concentration-Dependent Effect of Melatonin on the Core Pluripotency Transcription Factors in AD Fibroblasts

**Figure 5:**
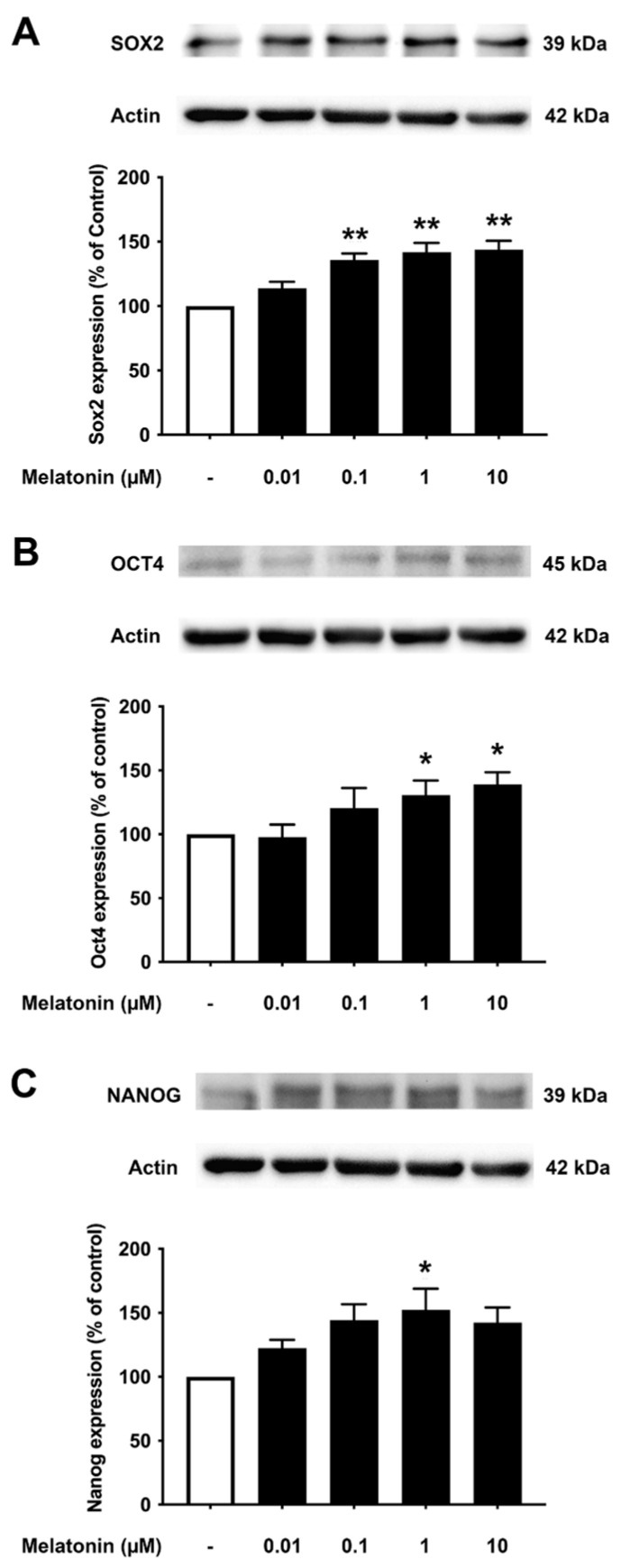
The effect of various concentrations of melatonin on (**A**) Sox2, (**B**) Oct4, and (**C**) Nanog expression levels in Alzheimer’s skin fibroblasts. Alzheimer’s skin fibroblasts were incubated in in various (0.01, 0.1, 1, 10 µM) concentrations of melatonin in serum-free media for 24 h. Control cells were cultured in serum-free media for 24 h. Western blotting was used for the analysis of protein expression levels with actin as a loading control. Results are reported as a percentage of the control. Data are expressed as mean ± SEM calculated using one-way ANOVA and Tukey’s post hoc test with significant difference at * = *p* < 0.05 and ** = *p* < 0.01 when compared to untreated controls (*n* = 3).

To examine the effect of different concentrations of melatonin on Sox2, Oct4, and Nanog in Alzheimer’s fibroblasts, cells were treated with varying concentrations of melatonin (0.01, 0.1, 1, 10 μM, and 0 μM for controls) for 24 h, and protein expression levels were determined using Western blotting. The results demonstrated that melatonin increased the expression of Sox2, Oct4, and Nanog in a concentration-dependent manner. Sox2 expression was shown to increase significantly at 0.1, 1, to 10 μM melatonin treatment compared to the untreated group. Oct4 expression levels were significantly increased at 1 and 10 μM melatonin treatment, while Nanog expression levels were significantly increased at 1 μM melatonin treatment compared to the untreated group (Figure 5A–C, Supplementary Figure S4).

### 3.6. Concentration-Dependent Effect of Melatonin on the Core Transcription Factors in SH-SY5Y Cells

**Figure 6:**
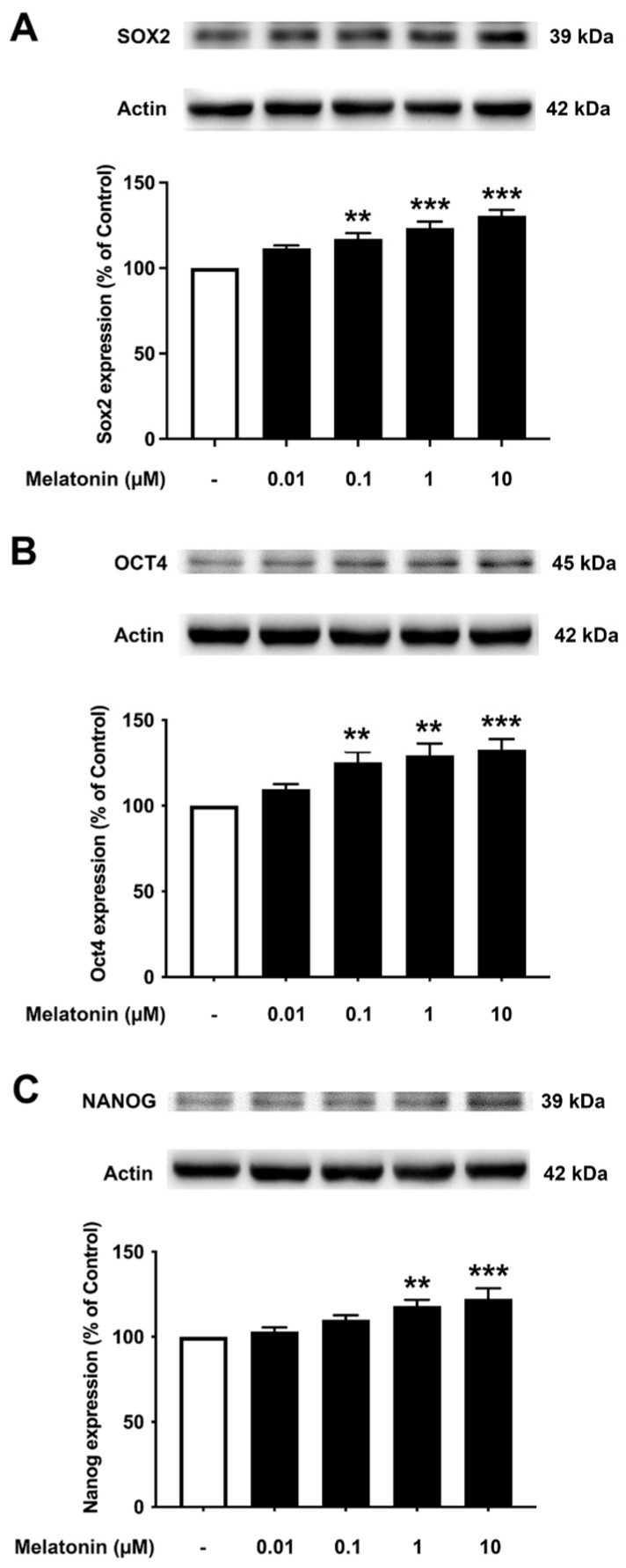
The effect of various concentrations of melatonin on (**A**) Sox2, (**B**) Oct4, and (**C**) Nanog expression levels in SH-SY5Y cells. SH-SY5Y cells were incubated in various concentrations (0.01, 0.1, 1, 10 µM) of melatonin for 24 h in serum-free media. Control cells were cultured in serum-free media for 24 h. Western blotting was used to analyze the expression of the protein with actin as a loading control. Results are expressed as a percentage of the control. Data are expressed as mean ± SEM with statistical significance at ** = *p* < 0.01 and *** = *p* < 0.001 when compared to untreated controls. Data are calculated using one-way ANOVA and Tukey’s post hoc test (*n* = 4).

To investigate the effect of different concentrations of melatonin on Sox2, Oct4, and Nanog expression levels in human neuroblastoma SH-SY5Y cells, cells were treated with varying concentrations of melatonin (0.01, 0.1, 1, 10 µM, and 0 µM for controls) for 24 h, and protein expression levels were determined using Western blotting. The results demonstrated that melatonin could increase the expression of Sox2 (Figure 6A, Supplementary Figure S5) significantly in a dose-dependent manner from 0.1, 1, and 10 µM along with Oct4 (Figure 6B), which was significant from 0.1, 1, and 10 µM compared to the control. The expression of Nanog (Figure 6C) was shown to be induced by melatonin significantly at 1 and 10 µM compared to the control group, respectively.

### 3.7. The Effect of Melatonin on the Expression Levels of the Core Transcription Factors Treated with Aβ42 in SH-SY5Y Cells

**Figure 7:**
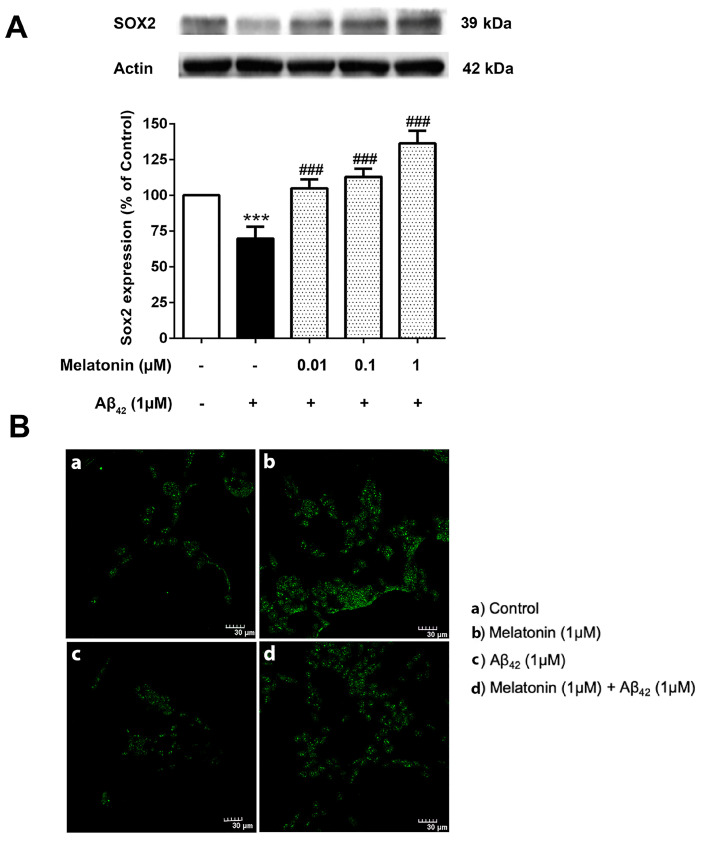
The effect of melatonin on the expression level of (**A**) Sox2 treated with Aβ42 in SH-SY5Y cells. Cells were pretreated with varying concentrations of melatonin (0.01, 0.1, 1 µM) for 2 h before being incubated with 1 µM of Aβ42 for another 24 h in serum-free media. Control cells were cultured in serum-free media for 24 h. Protein expression levels were analyzed using Western blotting with actin as a loading control. Results are expressed as a percentage of the control. Data are reported as mean ± SEM with significant difference at *** = *p* < 0.001 compared to untreated controls, and ### = *p* < 0.001 compared to Aβ42-treated groups. Data are calculated using one-way ANOVA and Tukey’s post hoc test (*n* = 4). (**B**). Immunostaining of SH-SY5Y cells demonstrating Sox2 expression (green). (**a**) Control cells were incubated in serum free media for 24 h. (**b**) Cells were treated with 1 µM melatonin alone for 24 h. (**c**) Cells were treated with 1 µM Aβ42 alone for 24 h. (**d**) Cells were pretreated with 1 µM melatonin for 2 h prior to 24 h 1 µM Aβ42 treatment. The green color indicates Sox2-positive immunostaining using Alexa 488-conjugated goat anti-rabbit IgG. Scale bar equals 30 μm.

The physiological imbalance of Sox2 levels plays a crucial role in the course of AD pathogenesis. It is then of interest to investigate the effect of Aβ42 on these transcription factor levels and whether melatonin pretreatment could alter their impact. SH-SY5Y cells were pretreated with 0.01, 0.1, and 1 µM of melatonin for 2 h prior to a 24 h 1 µM Aβ42 treatment. Western blot results demonstrated a significant reduction of Sox2 in 1 µM Aβ42 compared with controls and that melatonin pretreatment could increase the expression of Sox2 relative to the presence of Aβ42 alone at 0.01, 0.1 and 1 µM melatonin (Figure 7 and Supplementary Figure S6).

Immunostaining was conducted to visualize the difference in Sox2 expression in each condition (Figure 7B). The green Alexa 488-conjugated goat anti-rabbit IgG stain indicated a remarkably higher expression of Sox2 in the melatonin-treated group compared to the control, a lower expression of Sox2 in Aβ42 alone, and a visible increase in Sox2 in the Aβ42-treated group with prior exposure to melatonin treatment. The present result is consistent with Western blot analyses.

### 3.8. Effect of Luzindole (Melatonin Receptor Antagonist) on the Expression of Core Transcription Factors

**Figure 8:**
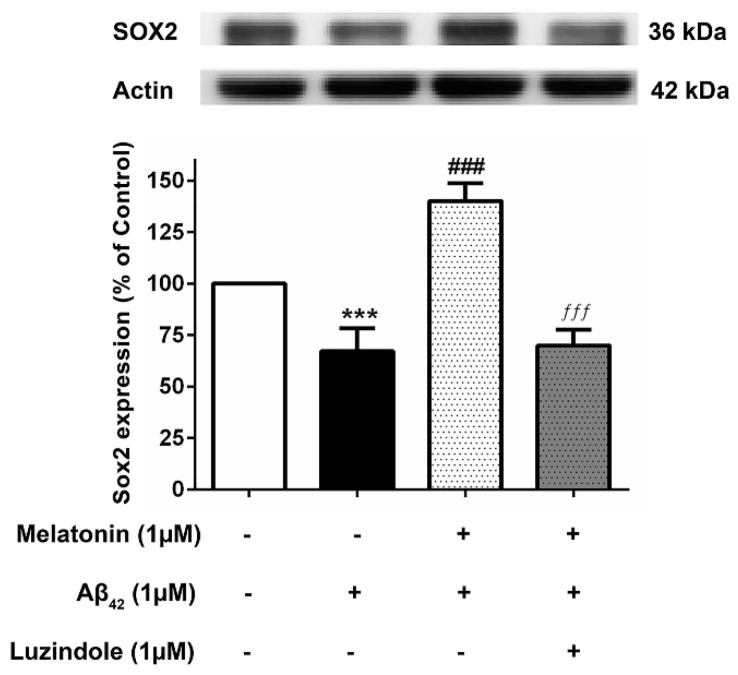
The effect of luzindole on Sox2 expression levels in the presence of melatonin and Aβ42. SH-SY5Y cells that were pretreated with 1µM melatonin and treated with 1 µM Aβ42 were incubated with 1 µM luzindole in serum-free media for 24 h and observed in comparison to the pretreatment group without luzindole, the 1 µM Aβ42-only treated group, and the control group cultured only in serum-free media for 24 h. Protein expression levels were then analyzed with Western blotting with actin as a loading control. Results are illustrated as a percentage of control. Data are expressed as mean ± SEM with statistical significance at *** = *p* < 0.001 compared to untreated controls, ### = *p* < 0.001 compared to the Aβ42-treated group, and ƒƒƒ = *p* < 0.001 compared to the melatonin pretreated without luzindole group. Data are calculated using one-way ANOVA and Tukey’s post hoc test (*n* = 4).

To investigate whether the action of melatonin in inducing these transcription factors is mediated through the melatonin receptor (MT1 and MT2), 1 µM luzindole (melatonin receptor antagonist) was administered together with the melatonin pretreatment group, and Sox2 expression levels were determined compared to their absence in the melatonin pretreatment and Aβ42 treatment group. The results illustrated that melatonin was ineffective in augmenting the expression of these factors in the presence of luzindole, indicating the involvement of the melatonin receptor in moderating the induction of these transcription factors (Figure 8), although other mechanisms cannot be ruled out.

**Figure 9:**
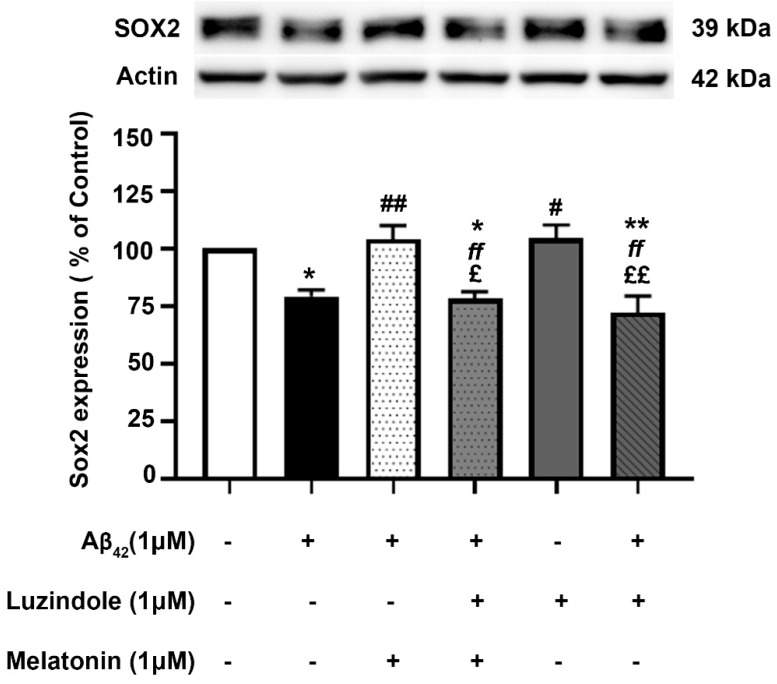
The effect of melatonin post-treatment and luzindole on Aβ42-induced changes in Sox2 expression in SH-SY5Y cells. SH-SY5Y cells were incubated at 37 °C with and without 1 µM Aβ42 for 30 min in serum-free media, followed by treatment with and without 1 µM luzindole for 30 min before 1 µM melatonin post-treatment for an additional 24 h. The analysis of Sox2 expression was performed by Western blot analysis. The band densities were normalized to actin. The ratios were calculated as a percentage of the respective value in the control (untreated) group. The data are expressed as the means ± SEM. One-way ANOVA and Tukey’s post-hoc test were performed for statistical analysis. (* and ** denote statistical significance at *p* < 0.05 and *p* < 0.01 compared to the control group, respectively; # and ## denote statistical significance at *p* < 0.05 and *p* < 0.01 compared to the group treated with Aβ42 alone, respectively; *ff* denotes statistical significance at *p* < 0.01 compared to the melatonin post-treatment group; and £ and *££* denote statistical significance at *p* < 0.05 and *p* < 0.01 compared to the group treated with luzindole alone).

The effect of post-treatment with melatonin on Aβ42-induced changes in Sox2 expression in SH-SY5Y cells was examined (Figure 9). SH-SY5Y cells were incubated at 37 °C with and without 1 µM Aβ42 for 30 min in serum-free media, followed by treatment with and without 1 µM melatonin for an additional 24 h. Western blot analysis of Sox2 expression was performed. The results showed that the expression of Sox2 in the group treated with 1 µM Aβ42 alone significantly decreased to 79.35 ± 2.735% compared to the control (untreated) group (*p* < 0.05). In the melatonin post-treatment group, Sox2 expression was significantly upregulated to 104.01 ± 5.917% compared to the group treated with 1 µM Aβ42 alone (*p* < 0.01), with no significant difference observed when compared to the control group. On comparing with the results of melatonin treated on AD fibroblasts, it could be well envisaged that melatonin could enhance the expression of Sox2 in AD fibroblasts. The present data suggested that melatonin is be able to restore the reduction of SOX2 expression in Aβ42-treated cells.

## 4. Discussion

In the present study, we evaluated the effect of melatonin administration on the expression of core transcription factors, Sox2, Oct4, and Nanog in young, aged, and AD patient fibroblasts. We also analyzed the expression of these factors in SH-SY5Y human neuronal cells where the professed expression trend was accorded in both experimental models. To affirm the characterization of fibroblasts, we first analyzed the basal expression levels of Sox2, Oct4, and Nanog. Fibroblasts prove to be an efficient model for the mechanistic study of AD pathology as they precisely present the cumulative cellular damage observed in patients. Our findings of the basal endogenous levels of Sox2, Oct4, and Nanog between the three fibroblast origins displayed a higher than two-fold decrease in Sox2 and Nanog levels in AD fibroblasts in comparison to the young and aged groups, while Oct4 levels similarly showed a declining pattern from young and aged to AD (Figure 2A–C). From the above findings, it can be well envisaged that the decrement in the endogenous levels of these transcription factors per se in aged and AD fibroblasts genuinely indicate the progressive pathology and could serve as promising biomarkers pertaining to senescence and neurodegenerative pathology.

Neurogenesis is dramatically decreased during aging [24], with significant dysregulation and reduction evidenced in both human AD brains and AD rodent models [25]. There have been ongoing debates and conflicting findings regarding adult hippocampal neurogenesis (AHN). However, increasing evidence supports the existence of AHN [26]. Adult neurogenesis is regulated and modulated by various extrinsic pathways, such as vascular, immune system, growth factor modulation, etc., and intrinsic pathways, which include epigenetic or genetic and signal transduction pathways, etc. Concerning the above-mentioned pathways, melatonin has been acknowledged for regulating and modulating such signaling pathways involved in both neurogenesis and neurodegeneration [22,23,27,28,29]. Interestingly, it has been predetermined that AD fibroblasts display APP processing, Aβ42, and tau pathology [30,31]. As melatonin has potential in alleviating and preventing the progression of AD [20,32] with its noteworthy role in stemness induction and improving reprogramming efficiency, as evidenced in mouse models, which probably involves the inhibition of the p53-mediated apoptotic pathway [33], we further investigated the expression of these transcription factors in response to melatonin treatment to identify the key elements, i.e., the possible capability of melatonin to enhance fibroblast reprogramming and an alternate pathway melatonin may utilize to prevent Aβ production and enhance neurogenesis.

It is noteworthy that apart from the rejuvenating properties of Sox2, it is also involved in interfering with the amyloidogenic processing of APP. Inhibition of α-secretase (ADAM10) activity reduces the proliferation of stem cell populations, as in neural progenitor cells (NPCs), mesenchymal stem cells (MSC) and human decidua parietalis placenta stem cells (hdPSC [34]), and its levels are significantly reduced in AD patients [35]. Our colleagues have established a functional interaction between Sox2 and APP, which is clearly evident from the transcriptional augmentation of ADAM10 by Sox2 [36]. The increased catalytic activity of ADAM10 and Sox2 expression is in conjunction with gamma (γ)-secretase inhibition, suggesting an antagonistic relationship between Sox2 and γ-secretase activity, demonstrated in both HEX 293 and human neuroblastoma SH-SY5Y cells lines [36]. The expression levels of Sox2 examined here were found to be consistent, with reported characteristics that indicate a strong reduction in the levels of pluripotency-related proteins, especially for Sox2, in the AD postmortem human brain [37] and mouse neuronal stem cells [38], which is most likely accompanied by impaired hippocampal neurogenesis in the early stages of AD as shown in Tg2576 (transgenic mice) resident and SVZ-derived adult neural stem cells [39]. Our present findings reveal that melatonin significantly enhanced the expression of Sox2 in young, aged, and AD fibroblasts (Figure 3A, Figure 4A and Figure 5A). In order to validate the findings from the fibroblast cells, we also examined the effect of melatonin treatment in human neuroblastoma SH-SY5Y cells, demonstrating that melatonin could increase the expression of Sox2 (Figure 6A) significantly in a concentration-dependent manner.

Aging leads to a decrease in tissue function and regenerative capacity. It has been demonstrated that the ectopic expression of Oct4 in retinal ganglion cells of aged mice is involved in promoting axonal regeneration and the restoration of youthful DNA methylation patterns and transcriptomes [40]. Moreover, an augmentation of Oct4 substantially increases Lamin B1 (a key regulator of DNA damage-induced senescence) and has thus been shown to exert anti-aging and anti-senescence effects in vascular cells of mice [18,41]. In consideration of such a critical role of Oct4, we further investigated whether melatonin exposure could alter its expression. Figure 3B, Figure 4B and Figure 5B clearly make evident that melatonin treatment determinately amplifies the expression of Oct4 in young, aged, and AD fibroblasts and SH-SY5Y cells, respectively (Figure 6B)

Taking into account the decisive functions of Nanog, it has been demonstrated that overexpression of Nanog enhances the potency of mesenchymal stem cells in order to develop into neural cells [42], and such overexpression of Nanog could possibly lead to the suppression of nuclear factor kappa-light-chain-enhancer of activated B cells (NF-κB) expression, a transcription factor that has a dominant role in inflammation and immune responses [43]. Additionally, Nanog increased proliferation and suppressed senescence in human primary fibroblasts, which is evidenced by a marked reduction in SA-β-gal activity, a marker widely used for senescence [44]. Chang CC and colleagues (2020) illustrated the protective role of Nanog where its upregulation in the rat brain mediates improvement in learning and memory along with attenuating the neurotoxic effects of Aβ [19]. The observation that melatonin dose-dependently and significantly boosted the expression of Nanog in all the three groups of fibroblasts (Figure 3C, Figure 4C and Figure 5C), and SH-SY5Y cells (Figure 6C), convincingly exemplifies melatonin function in regulating and enhancing the expression of Nanog. It has been previously reported that cellular prion protein (PrPc) regulates Nanog mRNA expression [45], and that melatonin regulates and modulates PrPc expression [46], which is clearly indicative of one of the many possible mechanisms of how melatonin augments Nanog expression, as demonstrated in our present study in both human fibroblasts and SH-SY5Y cells. To add on, miR-302 is predominantly expressed in iPSCs and is known to regulate several important biological processes of anti-oxidative stress, anti-apoptosis, and anti-aging through activating Akt signaling. miR-302-mediated Akt signaling further stimulates Nanog expression in order to suppress Aβ. In vivo studies have revealed that the mRNA expression levels of both Nanog and miR-302 were significantly reduced in AD patients’ blood cells [47]. Interestingly, melatonin-induced modulation of miR-302 variant increased the reprogramming efficiency of NSC-derived pluripotent stem cell (N-iPS) generation from primary cultured bovine NSCs mediated through the downregulation of p53 and p21 [48].

Concerning the other underlying molecular mechanisms of melatonin action in regulating these transcription factors, many more investigations are still required. Nonetheless, a few notable examples include the melatonin-induced regulation of Elk-1, a member of the ternary complex factors (TCFs) within the ETS (E26 transformation-specific) domain superfamily, a transcription factor implicated in neurodegeneration, neuroprotection, and brain tumor proliferation, which regulates Sox2, Oct4, and Nanog [49], and melatonin has been shown to substantially activate Elk-1 [50]. Sotthibundhu A and colleagues (2018) demonstrated that autophagy is involved in self-renewal by co-functioning with the ubiquitin-proteasome system (UPS) to promote Sox2, Oct4, and Nanog in human embryonic stem cells [51]. In this context, melatonin is well-known to regulate autophagy [52] and the UPS system [53], posing yet another pathway of controlling the expression of these transcription factors. In particular, our study revealed that melatonin is capable of augmenting and modulating the critical propellors of AD at a molecular level. Moreover, it has the potential to prevent senescence and aging characteristics.

**Figure 10:**
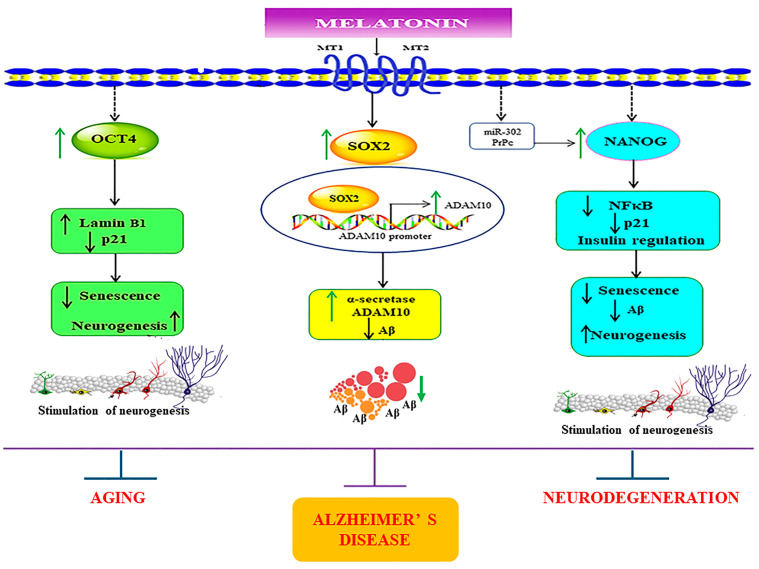
Schematic representation of the possible mechanisms underlying the melatonin-induced regulation of core transcription factors in SH-SY5Y and in young, aged, and Alzheimer’s skin fibroblasts. The figure demonstrates that melatonin amplifies the expression of the core transcription factors Sox2, Oct4, and Nanog. Firstly, melatonin via its receptor activation mediates its effects in enhancing the protein levels of Sox2. It has been previously established that the ADAM10 promoter has Sox2 binding sites, which indicates that melatonin, as a result, increases ADAM10 expression via Sox2-dependent transcriptional regulation. This simultaneously also aids in suppressing Aβ production. Secondly, melatonin-induced enhancement of Oct4 expression might lead to upregulation of Lamin B1 and downregulation of p21, which would be beneficial in diminishing senescent characteristics. Lastly, melatonin-dependent augmentation of Nanog might downregulate inflammation by plummeting NFκB expression and by modulating insulin signaling, preventing Aβ pathology. Additionally, melatonin might also regulate Nanog expression via modulating miR-302 and PrPc functions. Altogether, melatonin, by conforming and modulating these transcription factors, might effectively reduce senescence variations and pathological aging and provide therapeutic benefits for AD. ↑ = increase, ↓ = decrease.

In addition, we investigated the effect of Aβ_42_ administration on Sox2 expression in SH-SY5Y cells. A very significant reduction of Sox2 protein levels was observed (Figure 7A) corresponding to our findings in AD fibroblasts. Melatonin pretreatment restrained the detrimental effects of Aβ_42_ (Figure 6B). Complementing these findings, the immunocytochemical analysis revealed that melatonin pretreatment prevents Aβ_42_-induced alterations in Sox2 expression (Figure 7c). We further verified melatonin receptor involvement in the above experiment by using the melatonin receptor antagonist luzindole, indicating the likely involvement of the melatonin receptor in moderating the induction of these transcription factors, although other mechanisms cannot be ruled out (Figure 8 and Figure 9). We established, in these findings, the validation of human skin fibroblasts as a relevant model of AD and further revealed insights into melatonin’s potential in augmenting such pivotal transcription factors to enhance neurogenesis and prevent neurodegenerative pathology (Figure 10). Notably, the trend in the core pluripotent transcription factors Sox2, Oct4, and Nanog between all types of fibroblasts and the SH-SY5Y cells might provide insights into melatonin’s endogenous role in inducing stemness or factors relevant to the initiation of reprogramming. This is evident in studies demonstrating that the addition of melatonin to different reprogramming cocktails enhanced both the generation of iNSCs [48] through increased viability and the direct conversion of fibroblasts to neurons [23] via autophagy. Furthermore, melatonin was shown to selectively influence pluripotency and the differentiation of different cells at different concentrations [54]. Apart from its direct effect on Alzheimer’s pathway through the transcription factors, melatonin’s tendency to involve fibroblast reprogramming is briefly displayed in our study, which calls for a more profound investigation of the matter.

## 5. Conclusions

It is well established that melatonin levels decline with progressive aging, with a significant decline observed in AD patients. The ability of melatonin to upregulate Sox2, Oct4, and Nanog in normal and diseased conditions unravels its potential in enhancing neurogenesis and cell reprogramming in line with preventive and therapeutic approach towards aging and AD. Since these factors play a role in producing a degree of stemness that paves a stable base for both neurogenesis and cell fate determination in various types of cells, their pronounced deterioration in AD is undeniably suggestive of their therapeutic pertinency in the disease progression and validates the initial inspection of patient-derived fibroblasts for stemness and AD biomarker alteration where melatonin treatment suggests a better prognosis. Furthermore, in the future, we intend to establish concrete molecular links between these transcription factors pertaining to senescence, aging, and AD pathogenesis.

## Data Availability

Data are contained within the article.

## Supplementary Materials

The following supporting information can be downloaded at: https://www.mdpi.com/article/10.3390/biology13090698/s1, Figure S1. Full-length blots of Figure 2: Original photographs for the full length blots of the expression levels of Sox2, Oct4, and Nanog in young, aged, and Alzheimer’s skin fibroblasts of Figure 2A–C. Figure S2. Full-length blots of Figure 3: Original photographs for the full length blots of the effect of various concentrations of melatonin on (A) Sox2, (B) Oct4, and (C) Nanog expression levels in young skin fibroblasts of Figure 3A–C. Figure S3. Full-length blots of Figure 4: Original photographs for the full length blots of the effect of various concentrations of melatonin on (A) Sox2, (B) Oct4, and (C) Nanog expression levels in aged skin fibroblasts of Figure 4A–C. Figure S4. Full-length blots of Figure 5: Original photographs for the full length blots of the effect of various concentrations of melatonin on (A) Sox2, (B) Oct4, and (C) Nanog expression levels in Alzheimer’s skin fibroblasts of Figure 5A–C. Figure S5. Full-length blots of Figure 6: Original photographs for the full length blots of the effect of various concentrations of melatonin on (A) Sox2, (B) Oct4, and (C) Nanog expression levels in SH-SY5Y cells of Figure 6A–C. Figure S6. Full-length blots of Figure 7: The effect of melatonin on the expression level of Sox2 treated with Aβ42 in SH-SY5Y cells. Figure S7. Full blot of the effect of Luzindole, a melatonin receptor antagonist, on melatonin’s prevention of Aβ42-induced changes in Sox2 expression in SH-SY5Y cells. Figure S8. Full blot of melatonin post-treatment and Luzindole on Aβ42-induced changes in Sox2 expression in SH-SY5Y cells.

## Abbreviations

AD: Alzheimer’s disease; Aβ, amyloid beta; ADAM10, a disintegrin and metalloprotease; ETS, E26 transformation-specific; N-iPS, NSC-derived pluripotent stem cells; NF-κB, nuclear factor kappa-light-chain-enhancer of activated B cells; PrPc, prion protein; Sox2, SRY-Box transcription factor 2; TCFs, ternary complex factors; UPS, ubiquitin-proteasome system.
