# Supplementary material for: Melatonin Augments the Expression of Core Transcription Factors in Aged and Alzheimer’s Patient Skin Fibroblasts
Source: Biology (Basel). 2024 Sep 5;13(9):698. doi: 10.3390/biology13090698 (PMC11428320; doi:10.3390/biology13090698)
Supplement: Supplementary file 1 [file biology-13-00698-s001.zip › biology-3139306-supplementary.pdf]

Melatonin Augments the Expression of Core Transcription Factors in Aged and Alzheimer's Patient Skin Fibroblasts

Mayuri Shukla [1], Raphiporn Duangrat [1], Chutikorn Nopparat [2], Areechun Sotthibundhu [3] and Piyarat Govitrapong [1,*]

[1] Chulabhorn Graduate Institute, Chulabhorn Royal Academy, Kamphaeng Phet 6, Bangkok 10210, Thailand
[2] Innovative Learning Center, Srinakharinwirot University, Sukhumvit 23, Bangkok 10110, Thailand
[3] Chulabhorn International College of Medicine, Thammasat University, Pathumthani 12120, Thailand

Supplementary Figure S1:
Figure 2 (a, b, c)

Fig 2. Basal level comparison of Sox2, Oct4, and Nanog in young, aged, and Alzheimer's (AD) human primary dermal fibroblasts. Young, aged, and AD fibroblasts were cultured in serum free media at 37ºC for 24 h. Western blot analysis of a) Sox2, b) Oct4, and c) Nanog was performed. The band densities were normalized to actin. The ratios were calculated as a percentage of the respective value in the control (young) group. The data are expressed as the means ± S.E.M. One-way ANOVA and Tukey's post-hoc test were performed for statistical analysis. N = 3 (*, ** denote statistical significance at $p < 0.05$ and $p < 0.01$ compared to the control group, respectively).

figure 2a. Full blot of basal level of Sox2 in young, aged, and AD fibroblasts.

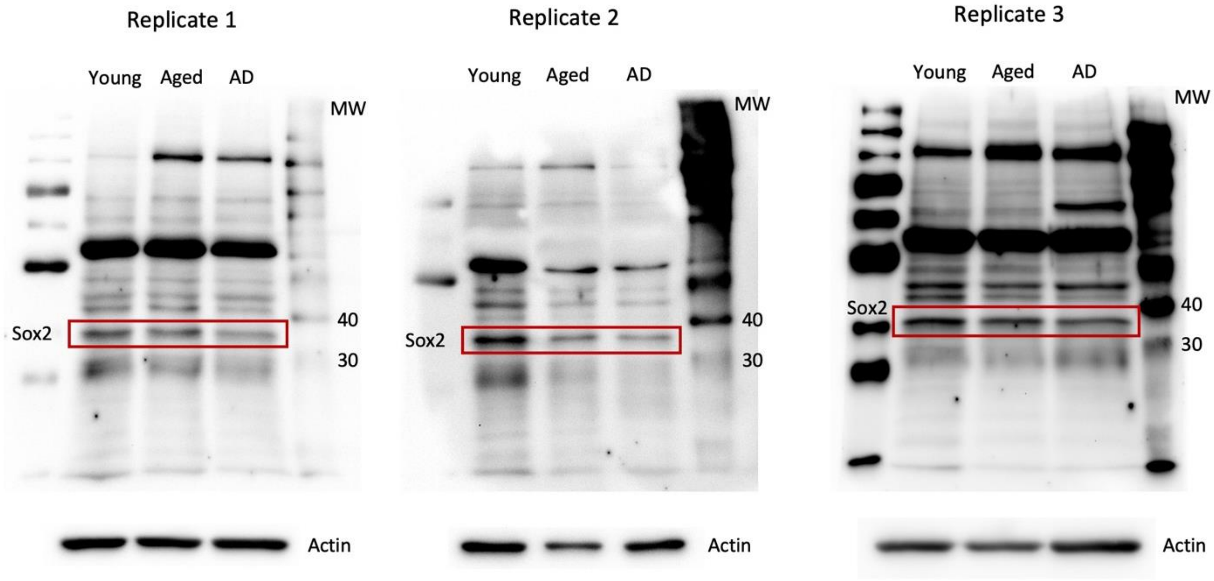

Supplementary figure 2b. Full blot of basal level of Oct4 in young, aged, and AD fibroblasts.

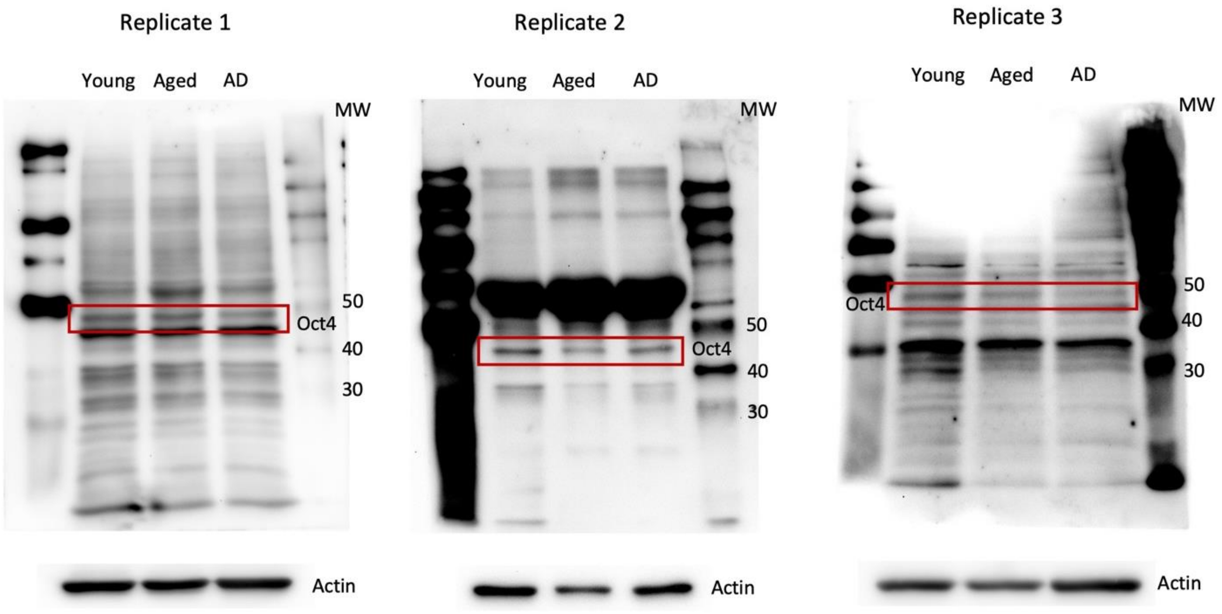

Supplementary figure 2c. Full blot of basal level of Nanog in young, aged, and AD fibroblasts.

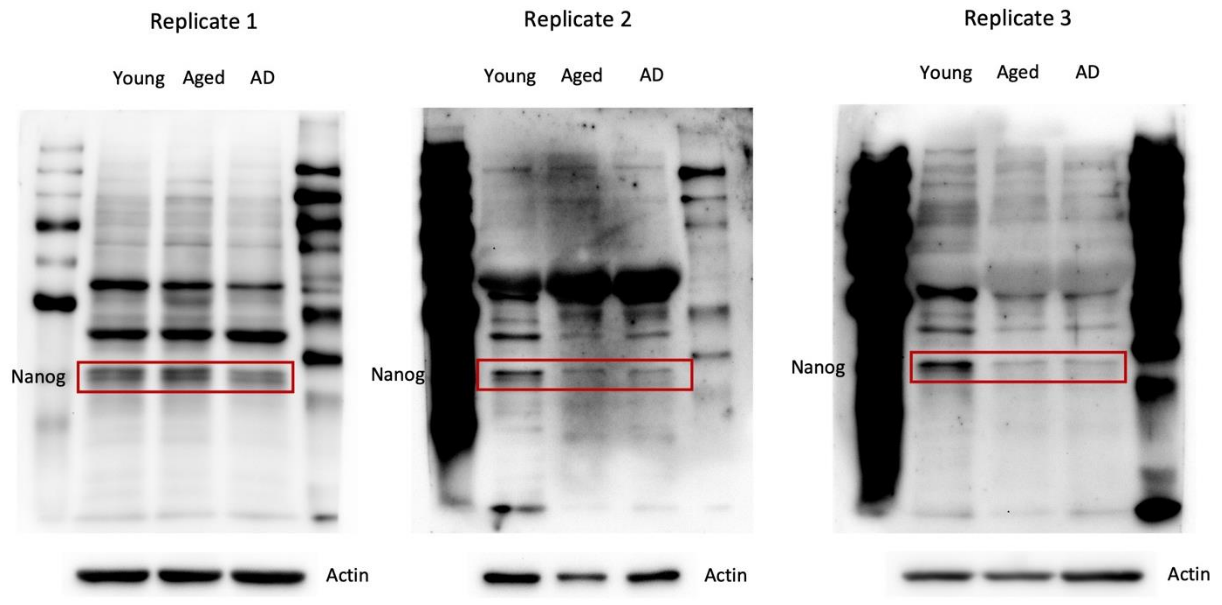

Supplementary Figure S2
figure 3 (a, b, c)

Fig 3. The effect of varying concentrations of melatonin on the pluripotency markers Sox2, Oct4, and Nanog in young fibroblasts. Young human primary dermal fibroblasts were incubated at 37ºC with 0, 0.01, 0.1, 1, and 10 µM of melatonin in serum free media for 24 h. Western blot analysis of a) Sox2, b) Oct4, and c) Nanog was performed. The band densities were normalized to actin. The ratios were calculated as a percentage of the respective value in the control (0 µM melatonin) group. The data are expressed as the means ± S.E.M. One-way ANOVA and Tukey's post-hoc test were performed for statistical analysis. N = 4 (*, **, and *** denote statistical significance at $p < 0.05$, $p < 0.01$, and $p < 0.001$ compared to the control group, respectively).

Supplementary figure 3a. Full blot of varying melatonin concentrations on Sox2 expression in young fibroblasts.

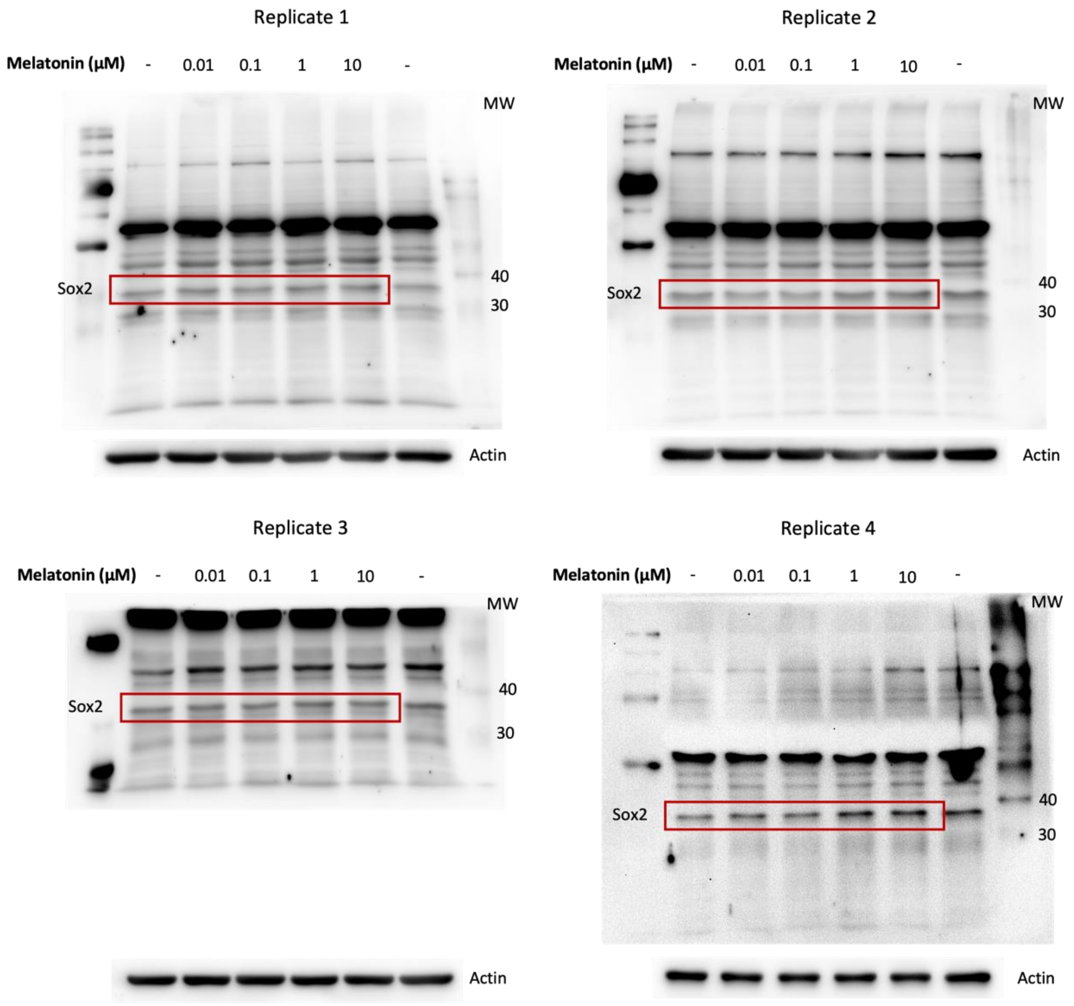

Supplementary figure 3b. Full blot of varying melatonin concentrations on Oct4 expression in young fibroblasts.

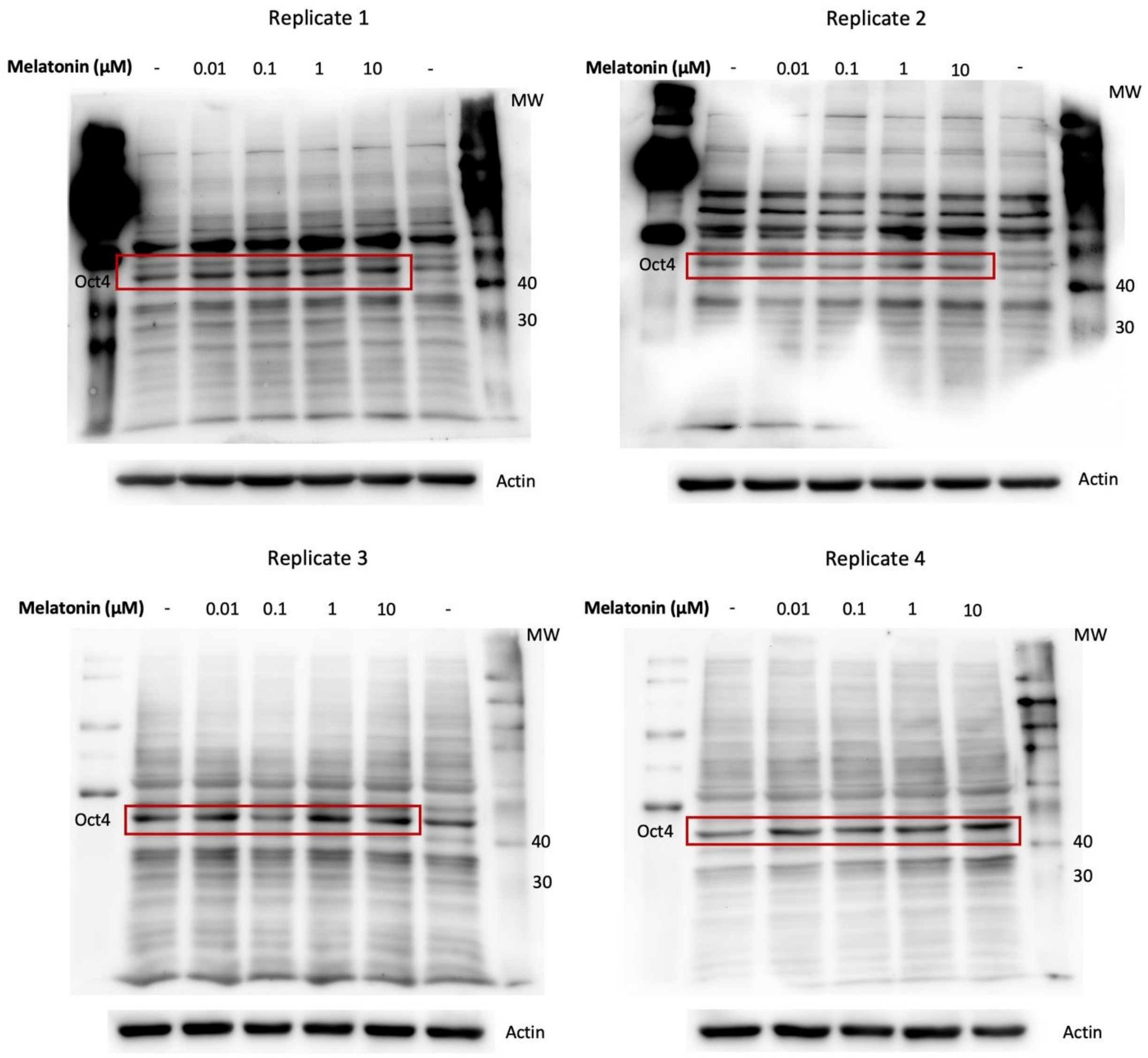

Supplementary figure 3c. Full blot of varying melatonin concentrations on Nanog expression in young fibroblasts.

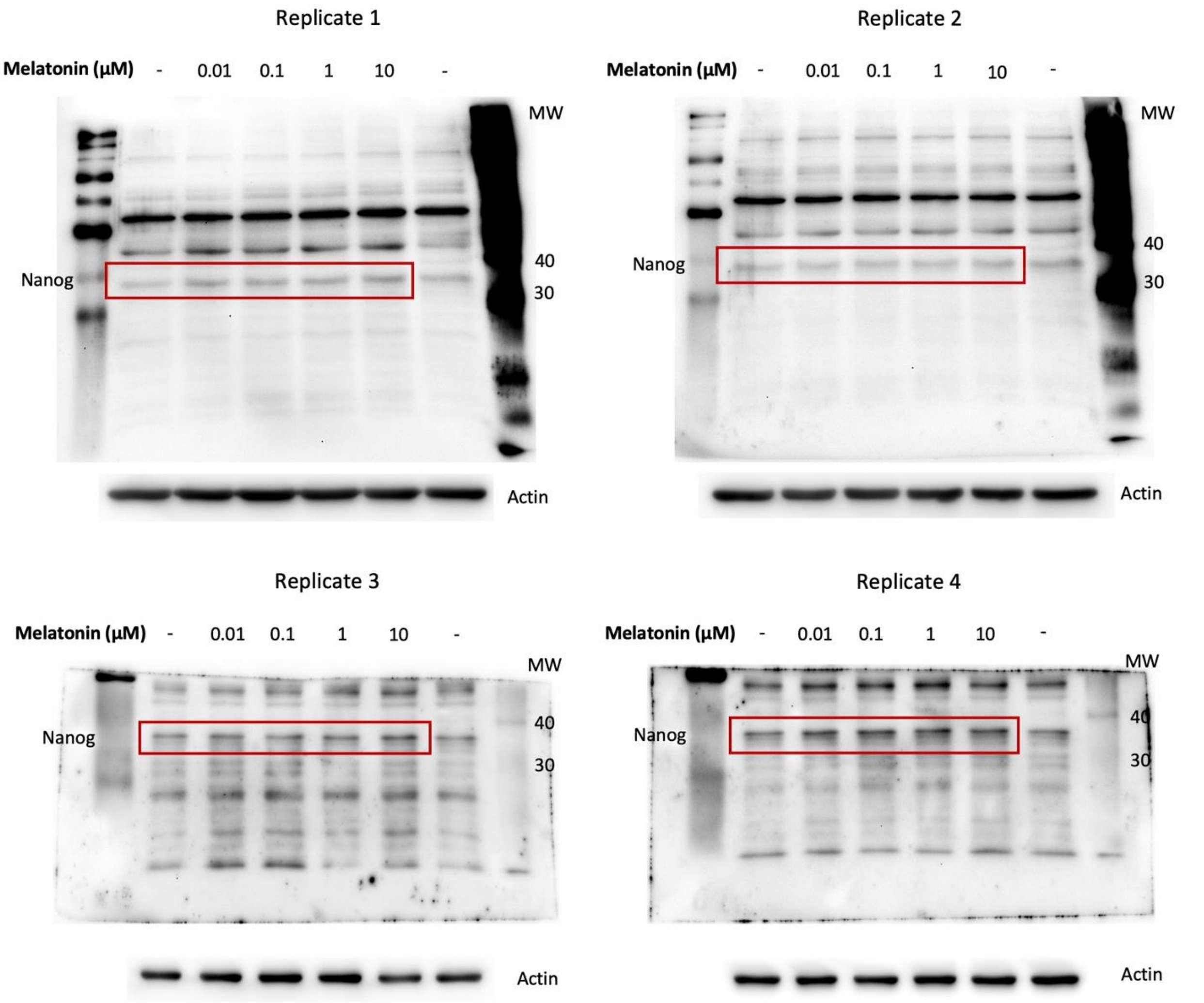

Supplementary Figure S3
figure 4 (a, b, c)

Fig 4. The effect of varying concentrations of melatonin on the pluripotency markers Sox2, Oct4, and Nanog in aged fibroblasts. Aged human primary dermal fibroblasts were incubated at 37ºC with 0, 0.01, 0.1, 1, and 10 μM of melatonin in serum free media for 24 h. Western blot analysis of a) Sox2, b) Oct4, and c) Nanog was performed. The band densities were normalized to actin. The ratios were calculated as a percentage of the respective value in the control (0 μM melatonin) group. The data are expressed as the means ± S.E.M. One-way ANOVA and Tukey's post-hoc test were performed for statistical analysis. N = 4 (*, **, and *** denote statistical significance at $p < 0.05$, $p < 0.01$, and $p < 0.001$ compared to the control group, respectively).

Supplementary figure 4a. Full blot of varying melatonin concentrations on Sox2 expression in aged fibroblasts.

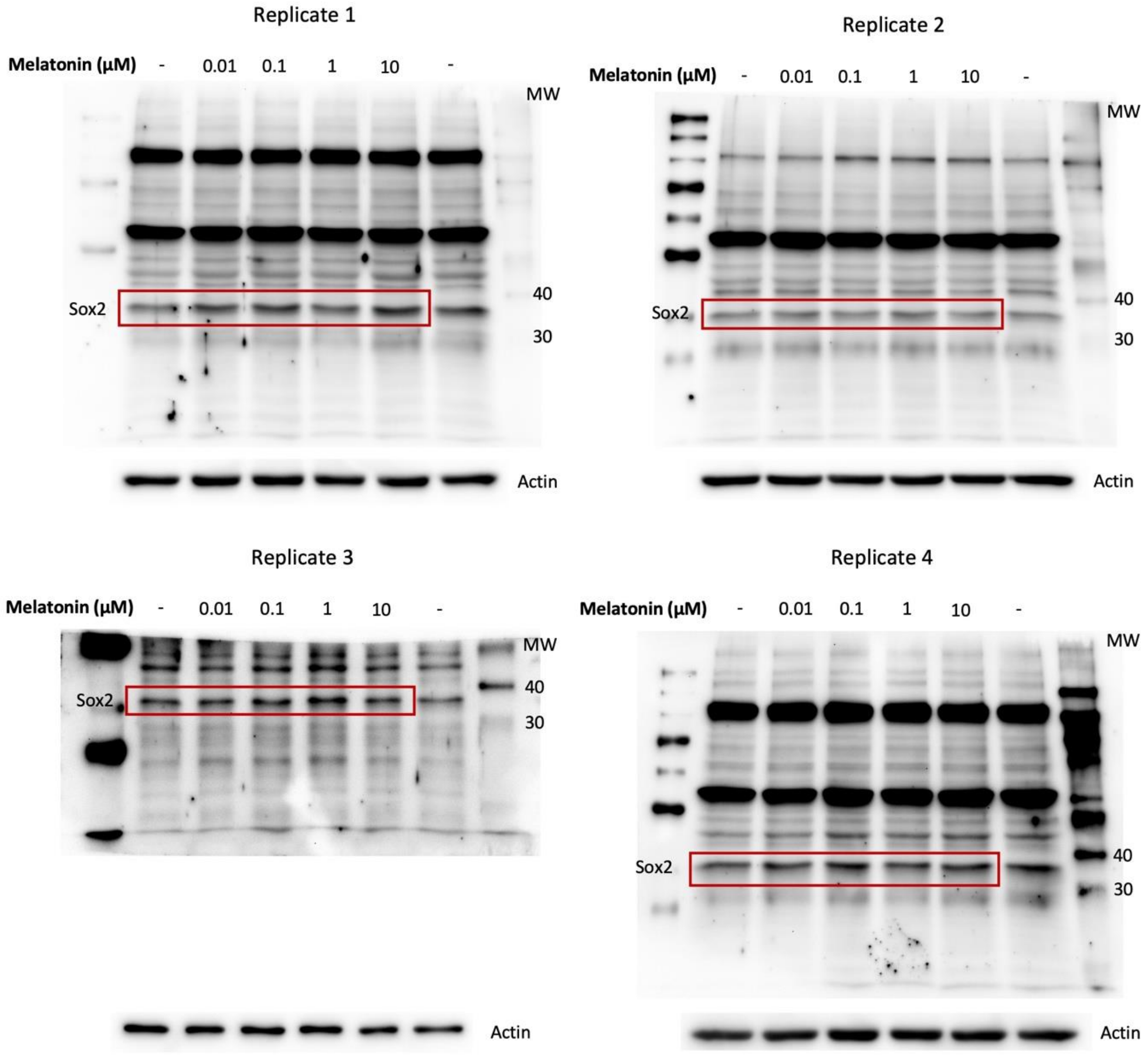

Supplementary figure 4b. Full blot of varying melatonin concentrations on Oct4 expression in aged fibroblasts.

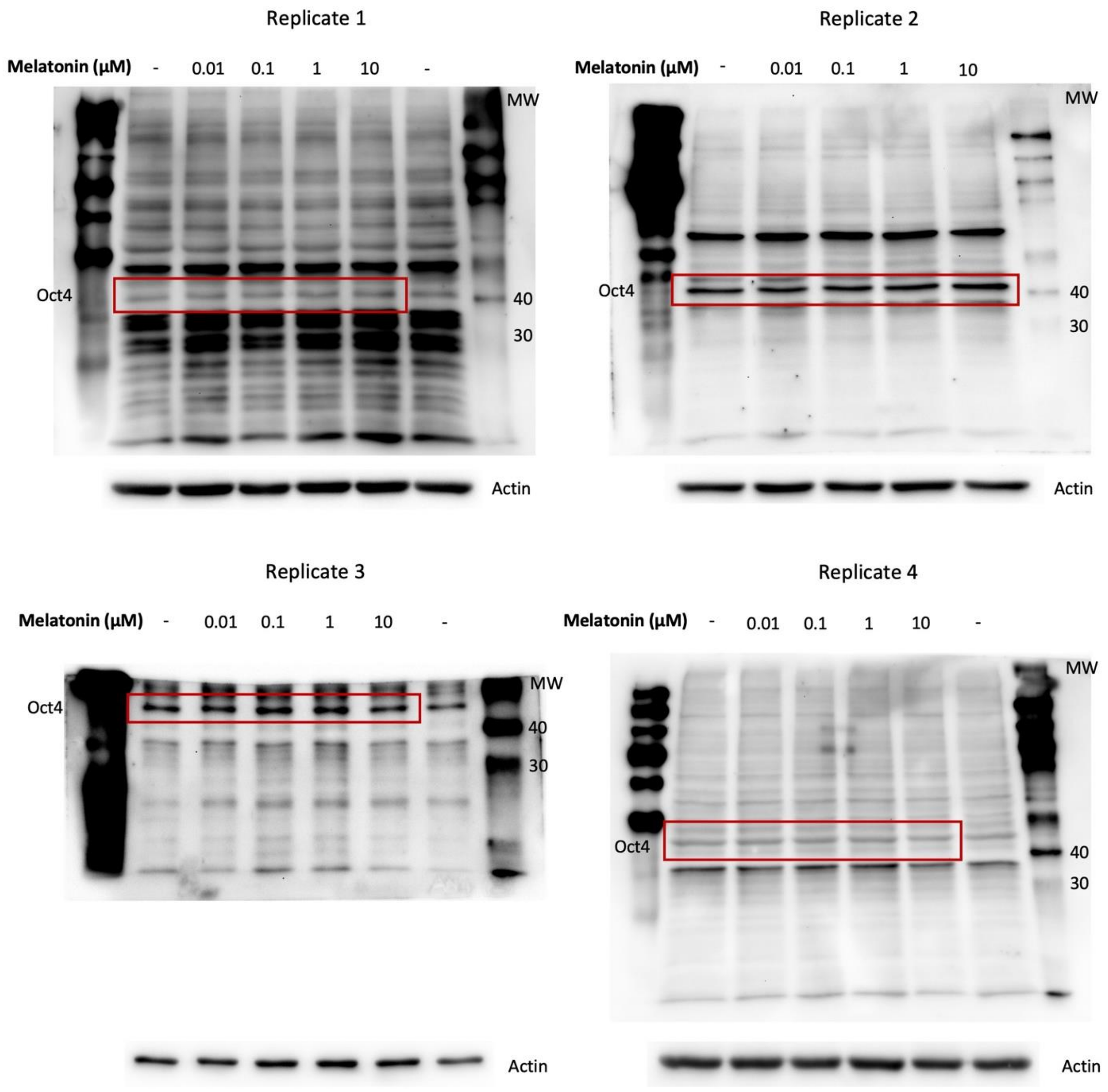

Supplementary figure 4c. Full blot of varying melatonin concentrations on Nanog expression in aged fibroblasts.

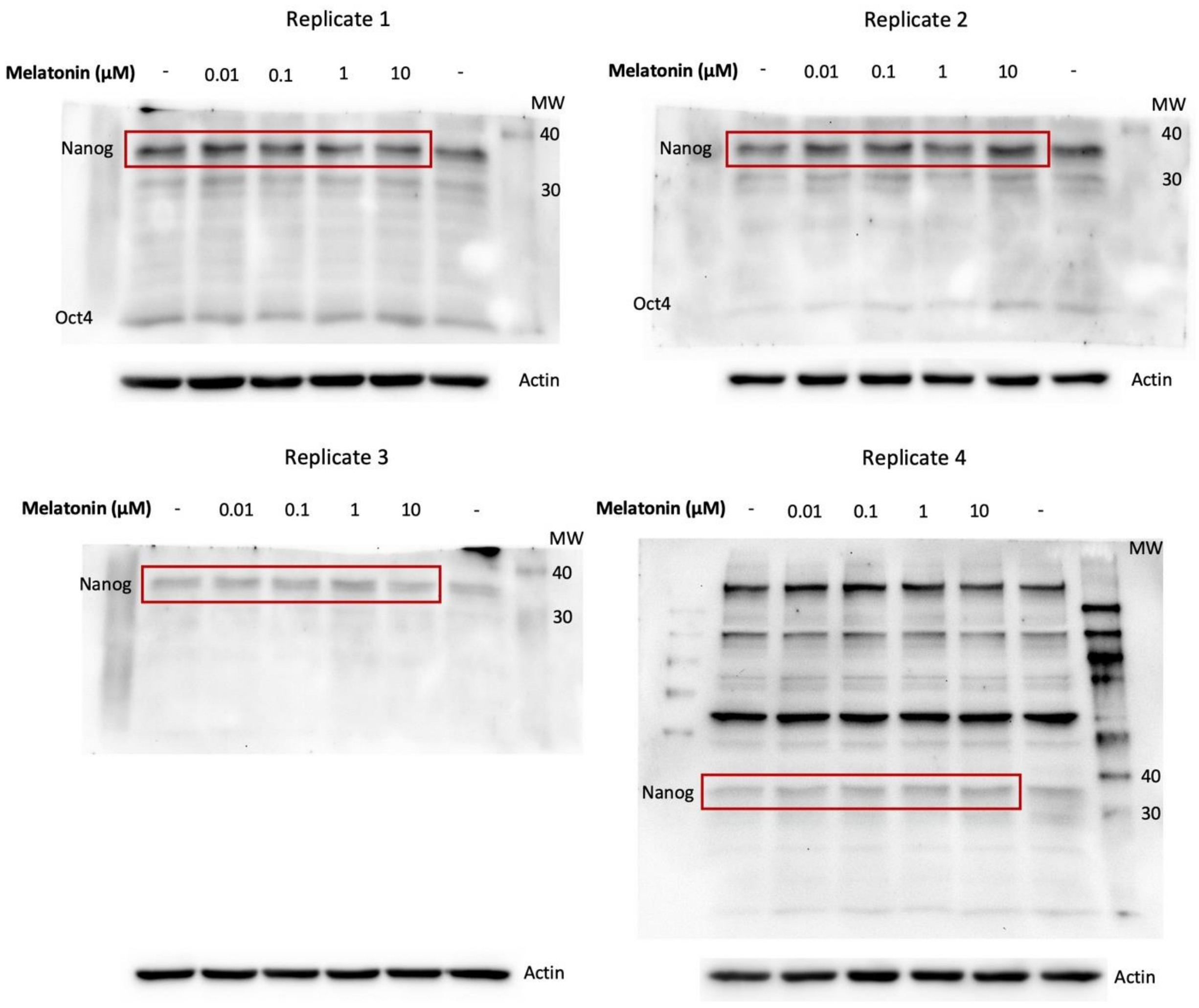

Supplementary Figure S4
figure 5 (a, b, c)

Fig 4. The effect of varying concentrations of melatonin on the pluripotency markers Sox2, Oct4, and Nanog in Alzheimer's (AD) fibroblasts. Alzheimer's human primary dermal fibroblasts were incubated at 37ºC with 0, 0.01, 0.1, 1, and 10 µM of melatonin in serum free media for 24 h. Western blot analysis of a) Sox2, b) Oct4, and c) Nanog was performed. The band densities were normalized to actin. The ratios were calculated as a percentage of the respective value in the control (0 µM melatonin) group. The data are expressed as the means ± S.E.M. One-way ANOVA and Tukey's post-hoc test were performed for statistical analysis. N = 3-4 (*, **, and *** denote statistical significance at $p < 0.05$, $p < 0.01$, and $p < 0.001$ compared to the control group, respectively).

Supplementary figure 5a. Full blot of varying melatonin concentrations on Sox2 expression in Alzheimer's (AD) fibroblasts. N = 3

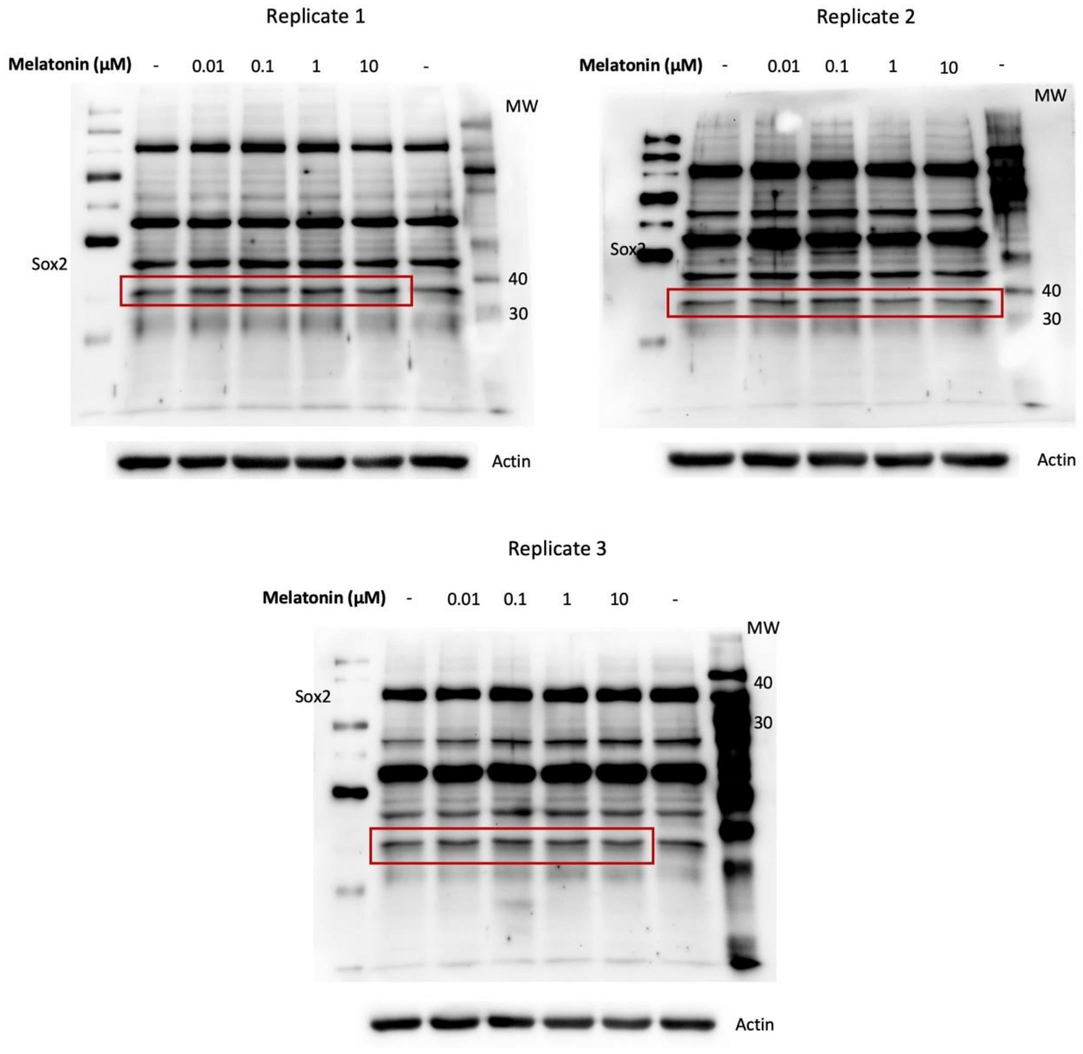

Supplementary figure 5b. Full blot of varying melatonin concentrations on Oct4 expression in Alzheimer's (AD) fibroblasts. N = 4

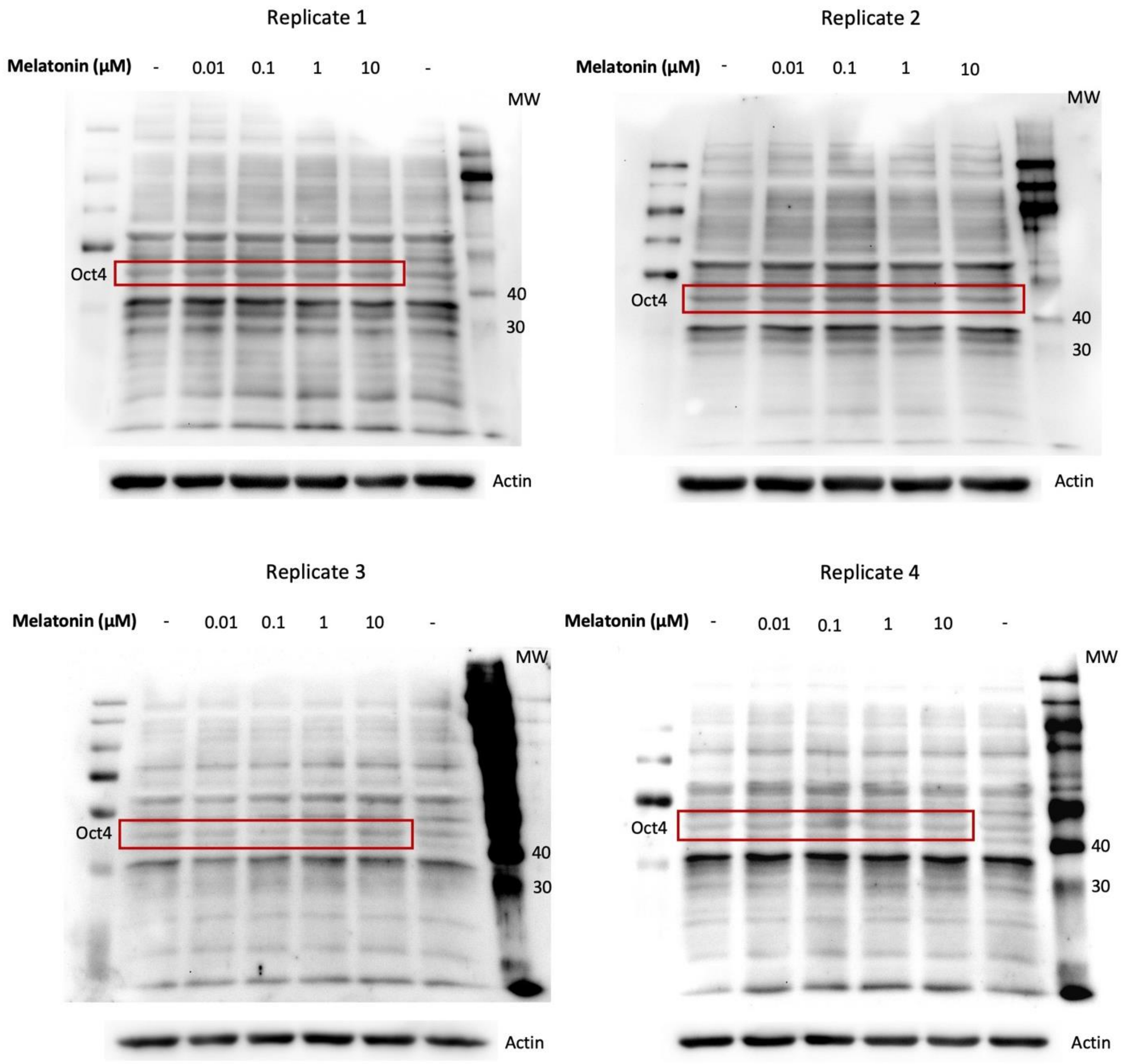

Supplementary figure 5c. Full blot of varying melatonin concentrations on Nanog expression in Alzheimer's (AD) fibroblasts. N = 3

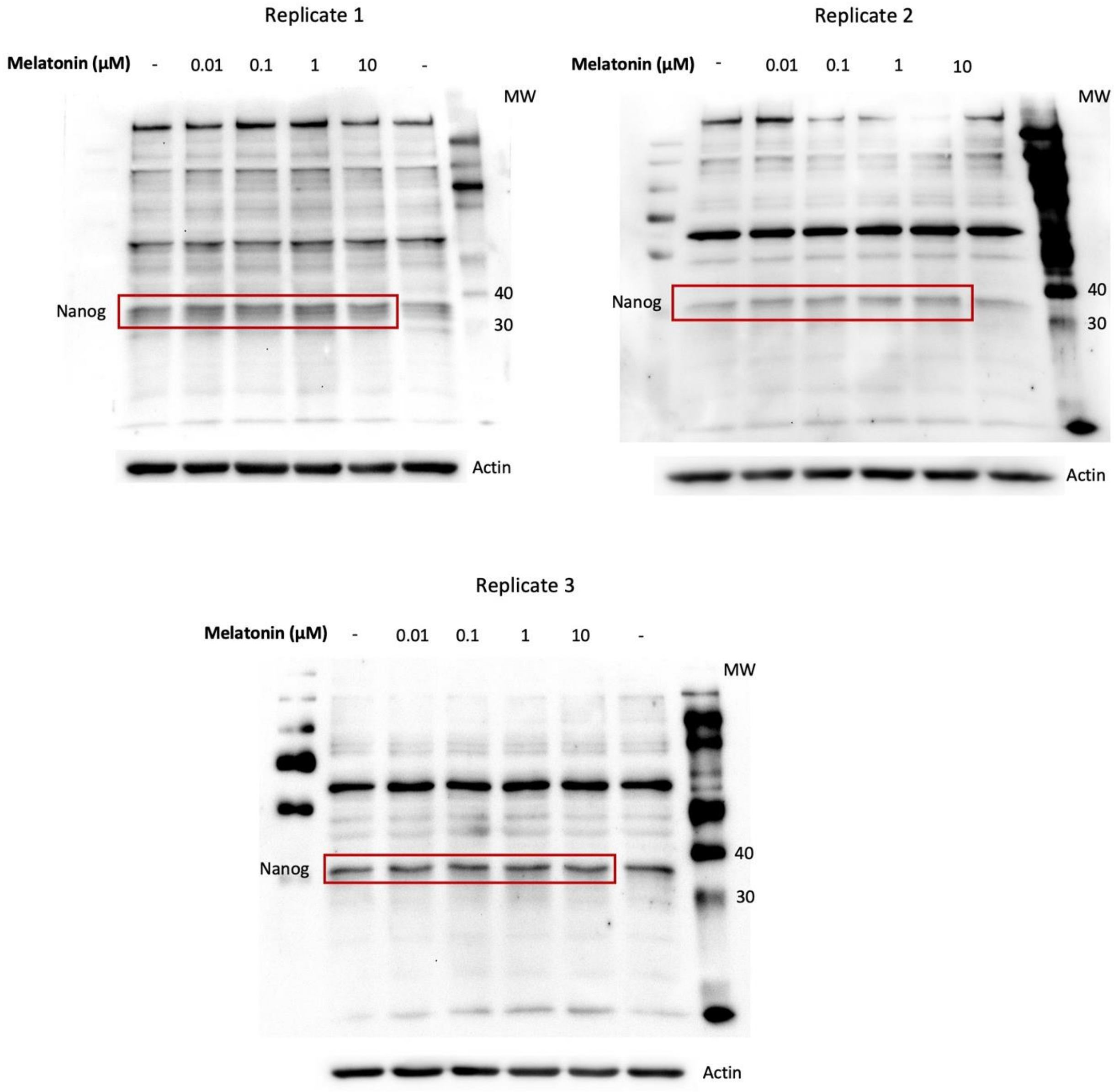

Supplementary Figure S5
figure 6 (a, b, c)

Fig 6. The effect of varying concentrations of melatonin on the pluripotency markers Sox2, Oct4, and Nanog in human neuroblastoma SH-SY5Y cells. SH-SY5Y cells were incubated at 37ºC with 0, 0.01, 0.1, 1, and 10 μM of melatonin in serum free media for 24 h. Western blot analysis of a) Sox2, b) Oct4, and c) Nanog was performed. The band densities were normalized to actin. The ratios were calculated as a percentage of the respective value in the control (0 μM melatonin) group. The data are expressed as the means ± S.E.M. One-way ANOVA and Tukey's post-hoc test were performed for statistical analysis. N = 4 (*, **, and *** denote statistical significance at $p < 0.05$, $p < 0.01$, and $p < 0.001$ compared to the control group, respectively).

Supplementary figure 6a. Full blot of varying melatonin concentrations on Sox2 expression in SH-SY5Y cells. N = 4

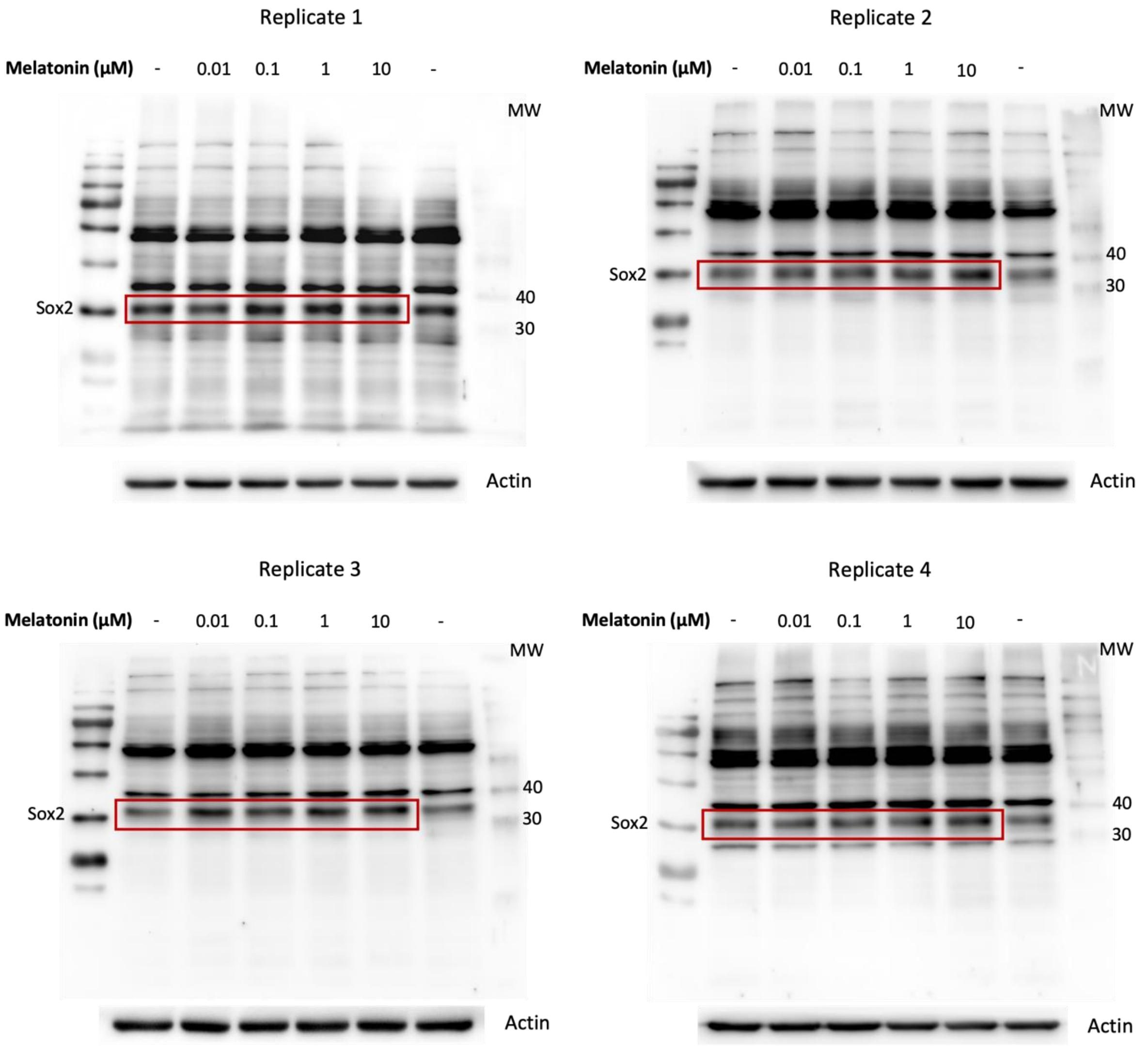

Supplementary figure 6b. Full blot of varying melatonin concentrations on Oct4 expression in SH-SY5Y cells. N = 4

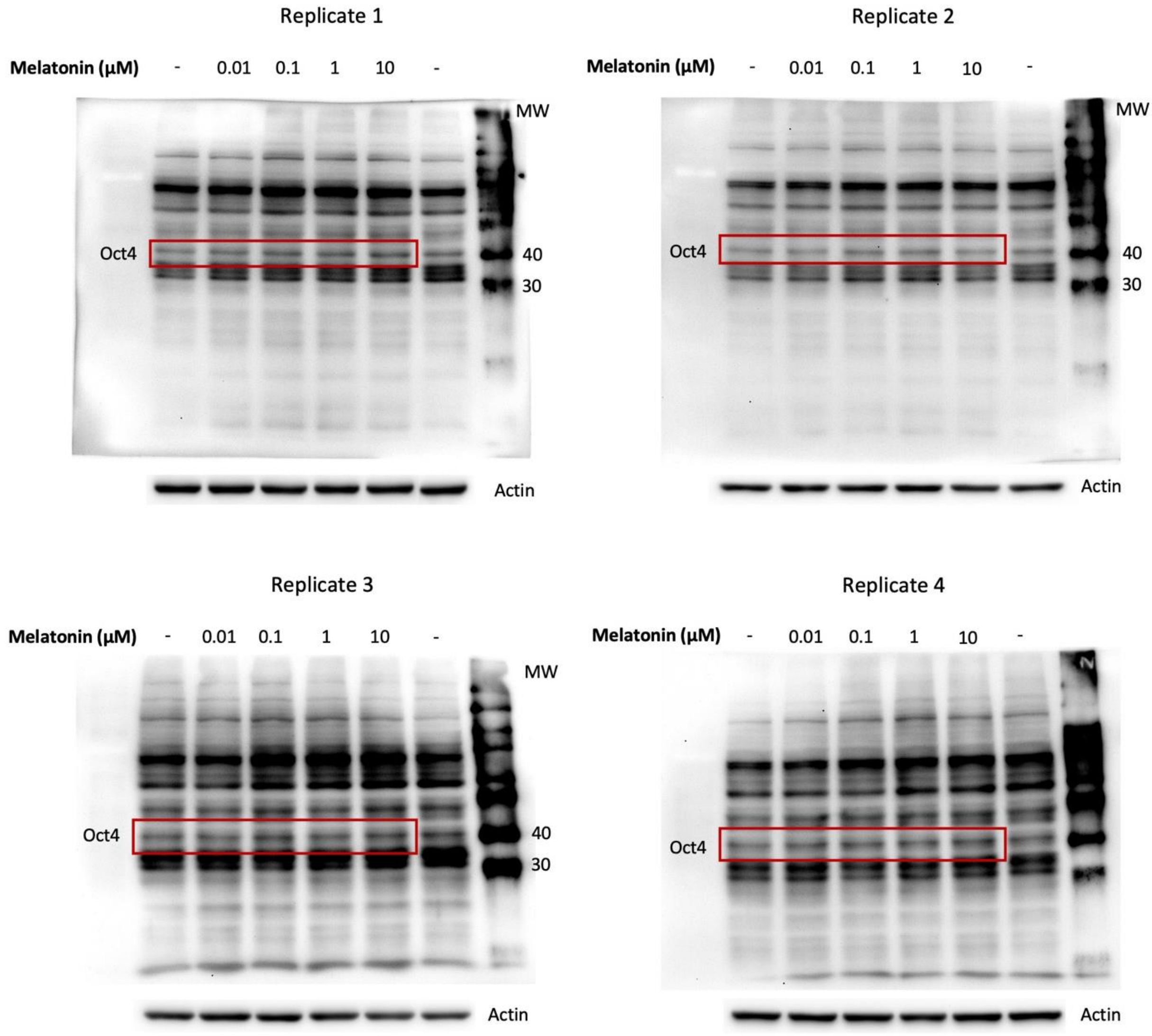

Supplementary figure 6c. Full blot of varying melatonin concentrations on Nanog expression in SH-SY5Y cells. N = 4

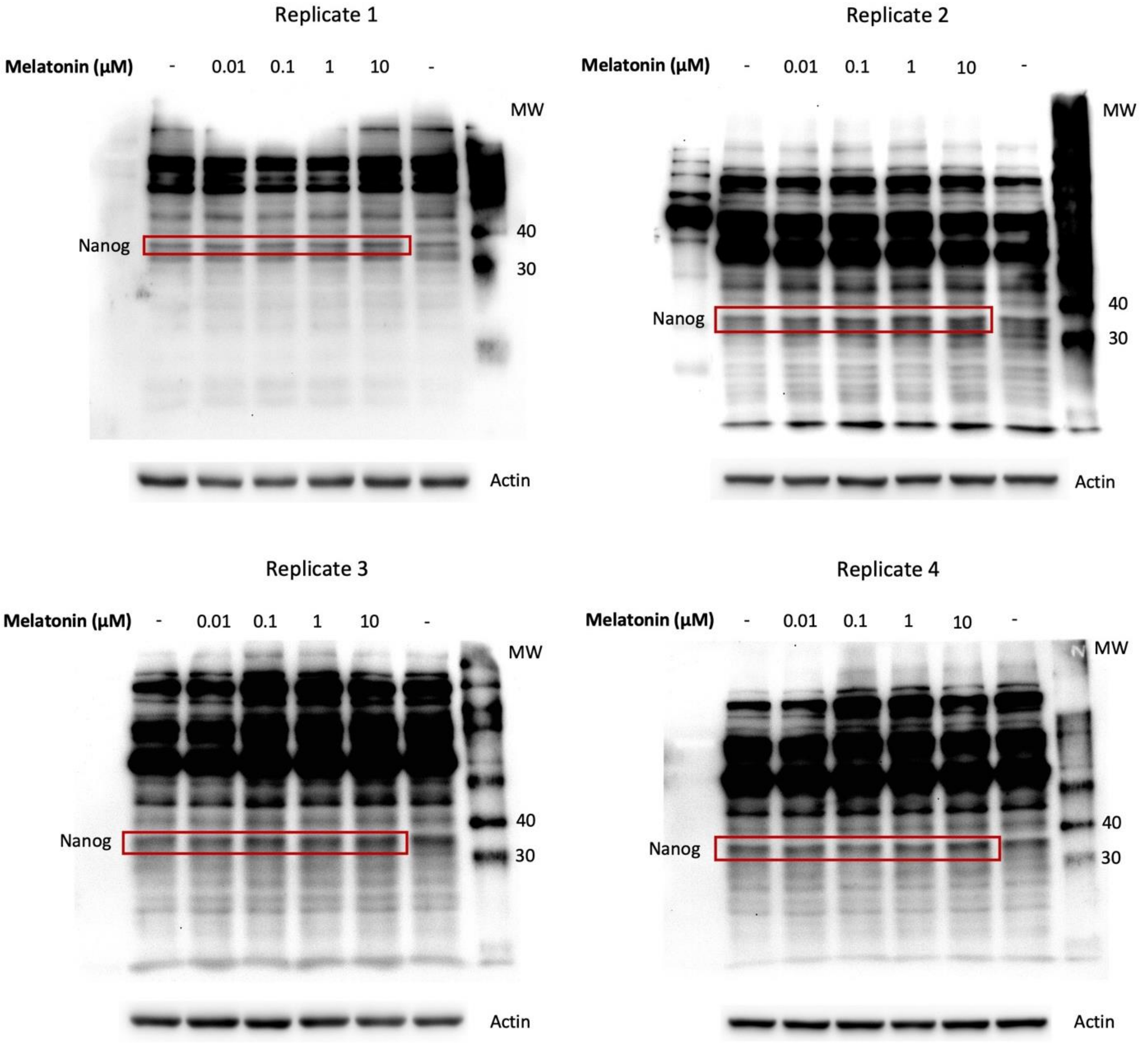

<u>Supplementary Figure S6</u>

<u>figure 7 (a)</u>

Fig 7. The effect of varying melatonin concentrations on Sox2 expression in SH-SY5Y cells treated with Aβ42. SH-SY5Y cells were incubated at 37ºC with 0, 0.01, 0.1, and 1 μM of melatonin for 2 h before treated with 1μM Aβ42 in serum free media for another 24 h. Western blot analysis of a) Sox2 was performed. The band densities were normalized to actin. The ratios were calculated as a percentage of the respective value in the control (untreated) group. The data are expressed as the means ± S.E.M. One-way ANOVA and Tukey's post-hoc test were performed for statistical analysis. N = 4 (*** and ### denote statistical significance at $p < 0.001$ compared to the control group, and Aβ42 group respectively).

Supplementary figure 7a. Full blot of varying melatonin concentrations on Sox2 expression in SH-SY5Y cells treated with $A\beta_{42}$. N = 4

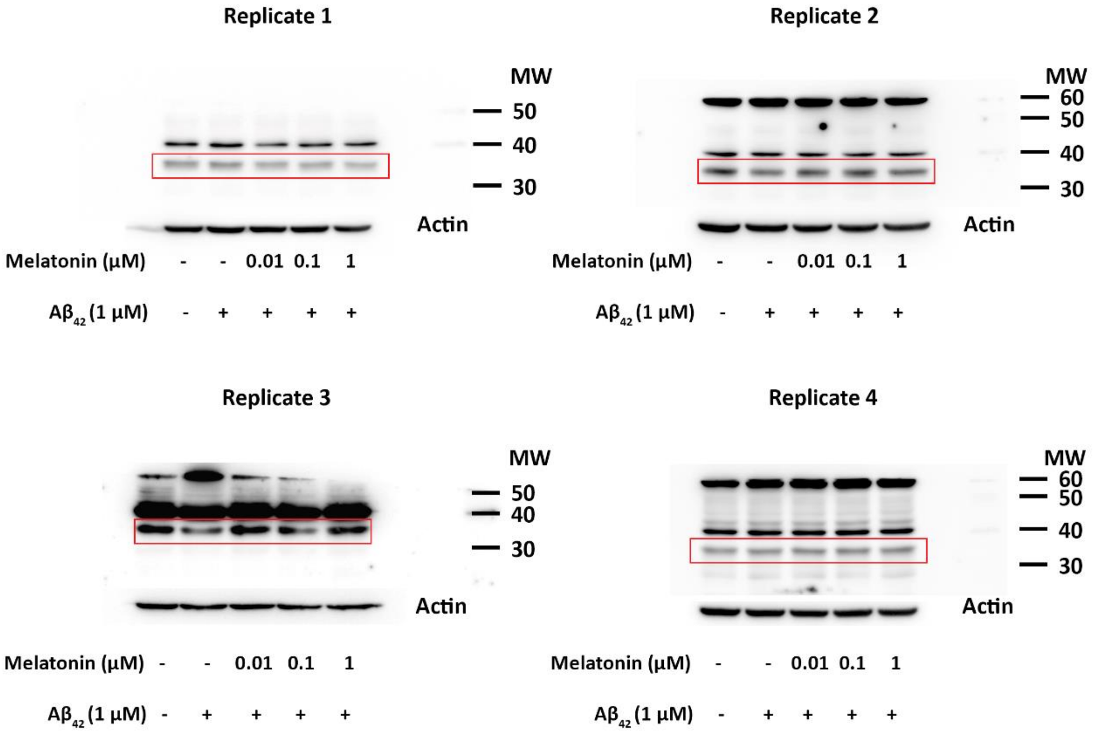

<u>Supplementary Figure S7</u>

Fig 8. The effect of Luzindole on pretreatement of melatonin on Aβ42-induced changes in Sox2 expression in SH-SY5Y cells. SH-SY5Y cells were incubated at 37ºC with 1 μM Luzindole for 30 min and followed by 1 μM melatonin for 2 h then 1 μM Aβ42 was given to the cells for 24 h in serum free media. Western blot analysis of Sox2 was performed. The band densities were normalized to actin. The ratios were calculated as a percentage of the respective value in the control (untreated) group. The data are expressed as the means ± S.E.M. One-way ANOVA and Tukey's post-hoc test were performed for statistical analysis. N = 4 (***, ###, and ∭ denote statistical

significance at $p < 0.001$ compared to the control group, Aβ42 group, and melatonin pretreatment group respectively).

Supplementary figure 8. Full blot of the effect of Luzindole, a melatonin receptor antagonist, on melatonin's prevention of Aβ42-induced changes in Sox2 expression in SH-SY5Y cells. N=4

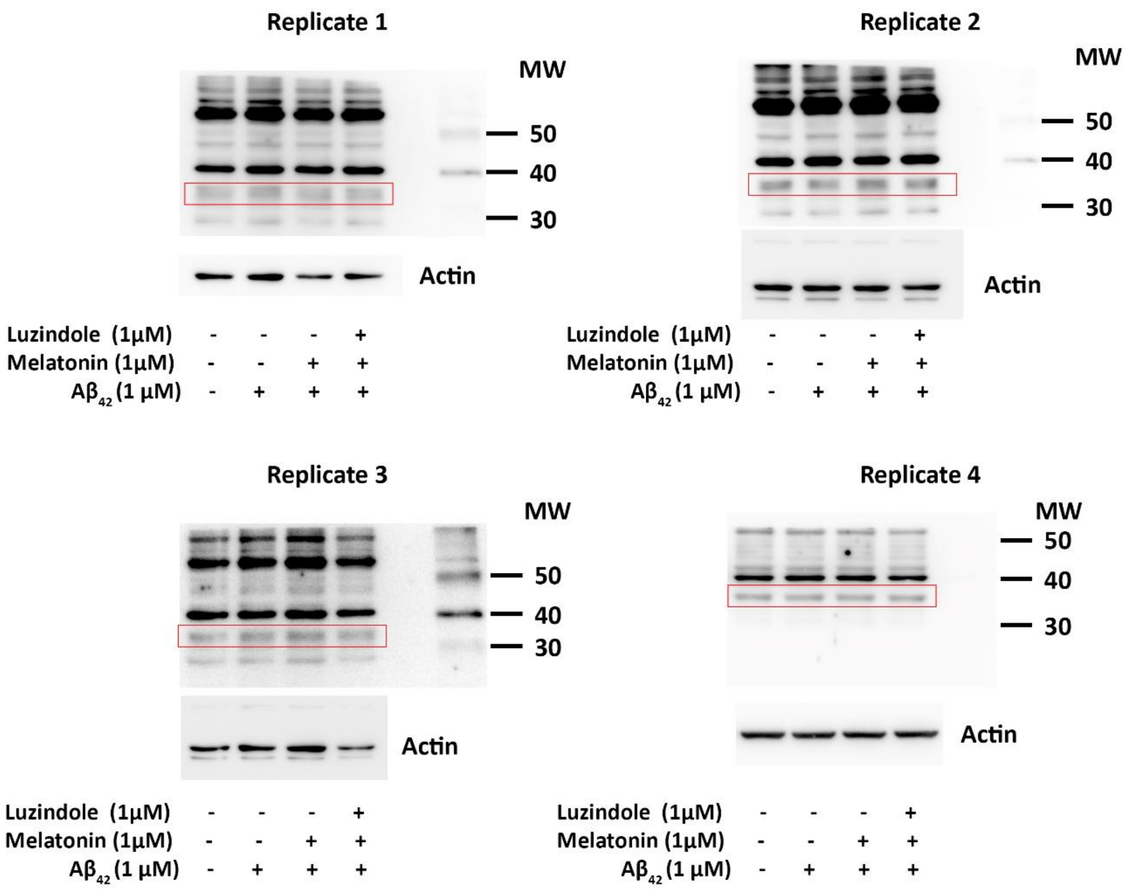

Supplementary Figure S8

Figure 9

Effect of melatonin post treatment and Luzindole on Aβ42-induced changes in Sox2 expression in SH-SY5Y cells. SH-SY5Y cells were incubated at 37°C with and without 1 µM Aβ42 for 30 minutes in serum-free media, followed by treated with and without 1 µM luzindole for 30 min before melatonin post-treatment for an additional 24 hours. The analysis of Sox2 expression was performed by western blot technique.The band densities were normalized to actin. The ratios were calculated as a percentage of the respective value in the control (untreated) group. The data are expressed as the means ± S.E.M. One-way ANOVA and Tukey's post-hoc test were performed for statistical analysis. (* and ** denote statistical significance at $p < 0.05$ and $p < 0.01$ respectively).

Supplement of full blot of melatonin post-treatment and Luzindole on Aβ42-induced changes in Sox2 expression in SH-SY5Y cells.

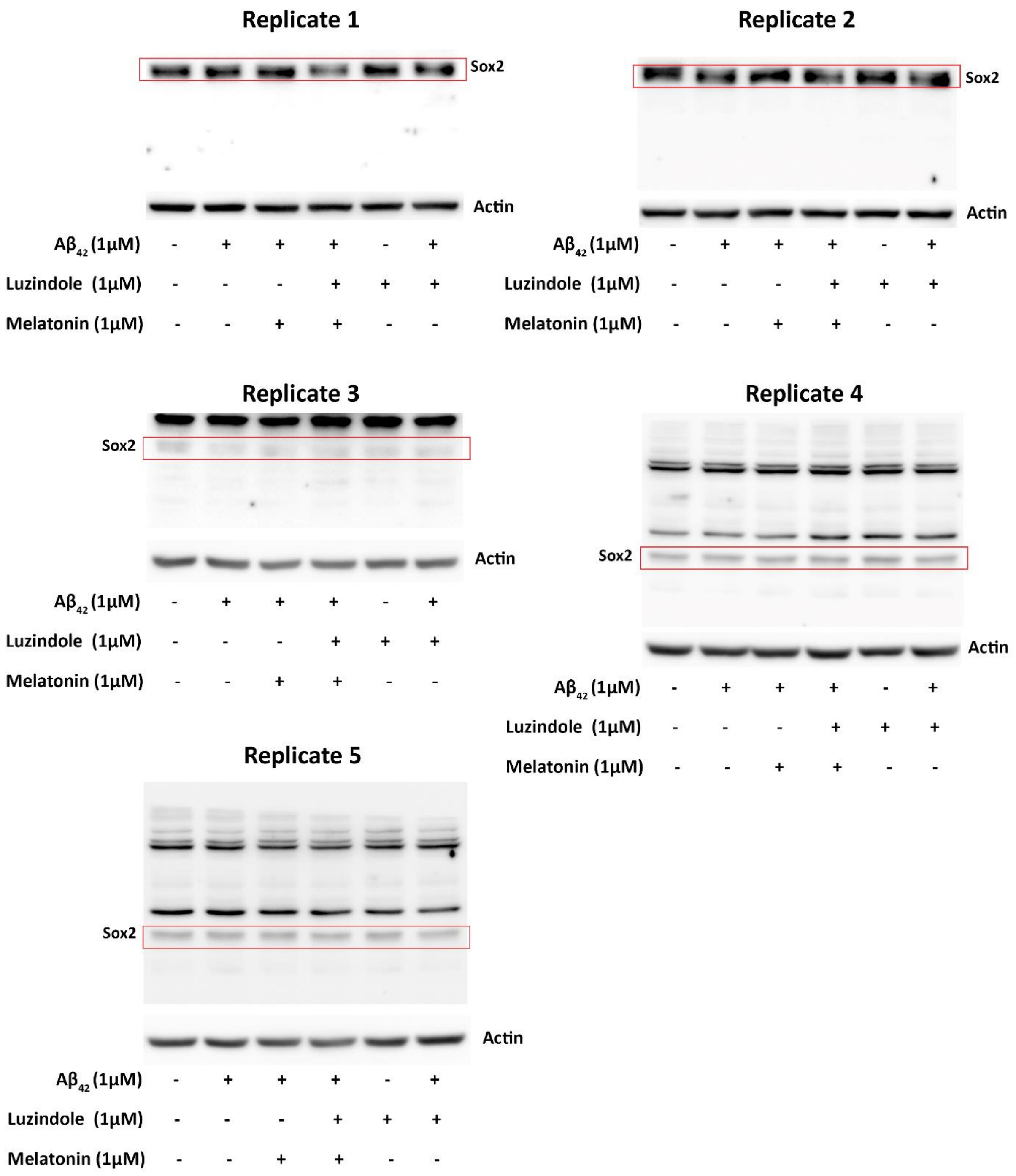
Replicate 1
Sox2
Actin
Aβ42 (1µM)
Luzindole (1µM)
Melatonin (1µM)
Replicate 2
Sox2
Actin
Aβ42 (1µM)
Luzindole (1µM)
Melatonin (1µM)
Replicate 3
Sox2
Actin
Aβ42 (1µM)
Luzindole (1µM)
Melatonin (1µM)
Replicate 4
Sox2
Actin
Aβ42 (1µM)
Luzindole (1µM)
Melatonin (1µM)
Replicate 5
Sox2
Actin
Aβ42 (1µM)
Luzindole (1µM)
Melatonin (1µM)
